# Primary Pyrrolimines and Pyridinimines

**DOI:** 10.3390/molecules30061239

**Published:** 2025-03-10

**Authors:** Amavi Kpoezoun, Gnon Baba, Jean-Claude Guillemin

**Affiliations:** 1 Univ Rennes, Ecole Nationale Supérieure de Chimie de Rennes, CNRS, ISCR (Institut des Sciences Chimiques de Rennes)—UMR6226, F-35000 Rennes, France; kpoezoun@gmail.com; 2 Laboratoire de Chimie Organique et des Substances Naturelles, Département de Chimie, Université de Lomé, Lomé BP 1515, Togo; gnonbaba@gmail.com

**Keywords:** pyrrolimine, pyridinimine, dehydrocyanation, retro-ene reaction

## Abstract

The association of an aromatic ring with an N-H-unsubstituted imine generates families of compounds that have been little studied until now except when the ring is a phenyl group. Recently, such imines substituted by a furan or thiophene group have been synthesized. This work reports a similar study where a pyrrole or pyridine ring is directly linked to an N-unsubstituted aldimine or ketimine group in order to isolate such compounds and to open the way to the knowledge of their physicochemical properties. The lower volatility of pyrrole and pyridine derivatives compared to aryl, furan, or thiophene derivatives greatly increases the difficulty of the synthesis and isolation of these kinetically unstable compounds.

## 1. Introduction

The synthesis of new unstabilized imines has been a challenging subject for many decades. The simplest, CH_2_=NH, was synthesized by a retro-Diels–Alder reaction from an aza-norbornene derivative [1], the dehydrochlorination of N-chloromethylamine [1,2], thermolysis [3], or the photolysis of methyl azide [4] and later by the dehydrocyanation of aminoacetonitrile [5]. Such reactions have been extended to the synthesis of C-monosubstituted derivatives, the N-unsubstituted aldimines, but many with an alkyl substituent [2,5]. On the other hand, the retroenic reaction of secondary allyl- and propargyl-amines provided the expected imine and propene or allene, respectively, opening the way to vinyl- or ethynylimine [6,7]. Thus, very recently, the thermolysis of dipropargylamine followed by the recording of the millimeter wave spectrum of the ethynylimine formed allowed its detection in the interstellar medium (ISM) [7] where methanimine [8], ethanimine [9], cyanomethanimine [10,11], and N-cyanomethanimine [12] have also been observed. On the other hand, the photolysis of an azide deposited on a cold window allowed the first detection by infrared spectroscopy of a functionalized N-H-aldimine, HCOCH=NH [13], or of a diamine, 1,4-diazabutadiene, HN=CH-CH=NH [14]. In a recent article on the theoretical chemistry of candidates for the ISM, it was interesting to see the large number of imines of formula CmHnN_2_ that have never been synthesized [15].

In our approach, where the isolation and chemistry of the synthesized compounds are essential parameters, the preparation of the simplest derivatives of a family of compounds associated with a possible selective addition of substituent(s) allows us to obtain good knowledge of these target species. We have recently reported the syntheses of two imines, HCN dimers, methylenecyanamide (CH_2_=NCN) and (Z)- and (E)-Iminoacetonitriles (NC-CH=NH) [16], and of the derivatives of two new families of N-H aldimines, one substituted by a furan group [17] and the second by a thiophene group [18]. All these compounds were obtained in gas phase, paving the way for their characterization by many techniques (millimetric, infrared or UV-VIS in the gas phase, photoelectron spectroscopy, gas electron diffraction, mass spectrometry). For all synthesized derivatives bearing a heterocyclopentadiene substituent, and even for the most reactive unsubstituted derivatives, transimination reactions were efficiently carried out showing the potential of such compounds as precursors of many imines. However, compared to simple N-H aldimines, we observed an increase in the difficulty of the gas phase transfer of these imines after the reactor or the furnace. We have drawn an analogy with the atmospheric pressure boiling point of furan (31.5 °C) and thiophene (84 °C) and the synthesized imines that are really different, e.g., from the highly volatile C3 imines with the boiling points of ethane (−88.6 °C), ethene (−103.9 °C), or ethyne (−84.7 °C) for comparison. In the literature, the most studied heterocyclopentadienes are unambiguously the furan, thiophene, and pyrrole derivatives. Thus, this study focused on the synthesis, isolation, and chemistry of the third one leading to the pyrrolimines. For pyrrole, a boiling point of 129.7 °C has been reported indicating a probably high difficulty in obtaining pyrrolimines in the gas phase. Due to the trivalent nitrogen atom, a six-membered aromatic ring, pyridine is also a possible substituent. So, we also studied pyridinimines, a family of compounds with a pyridine ring as an aromatic substituent.

**Scheme 1:**
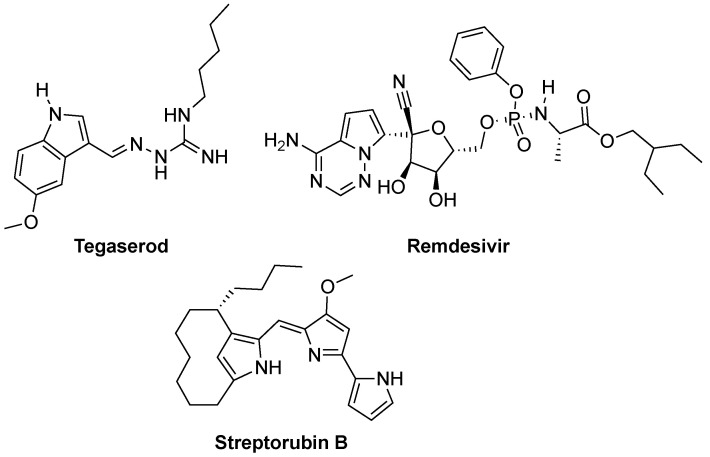
Drugs with a pyrrolimine motif.

Pyrrolimines, as well as furanimines [17] and thiophenimines [18], are of great interest as bioactive and pharmaceutical compounds. As with drugs containing the thiophenimine moiety, the pyrrolimine moiety is often found in polycyclic systems where the C=N group is part of a pyrrole, pyrimidine, or pyridazine ring. For example, Remdesivir [19], Baricitinib [20], prodiginines [21] (prodigiosin R1, metacycloprodigiosin, undecylprodigiosin, roseophilin, streptorubin B) are therefore commercial drugs formally containing pyrrolimine moieties (Scheme 1). Remdesivir, marketed as Veklury, is a broad-spectrum antiviral agent. It has demonstrated efficacy against a broad range of viruses [22], including filoviruses such as Ebola and Marburg, and coronaviruses such as SARS-CoV, MERS-CoV, and SARS-CoV-2 [23,24]. In addition, it was the first antiviral to be shown to be effective and approved for the treatment of coronavirus disease 2019 (COVID-19) [25,26,27]. Tegaserod, on the other hand, has an acyclic imine motif associated with pyrrole. It is a drug used for the treatment of irritable bowel syndrome (IBS) [28,29].

**Scheme 2:**
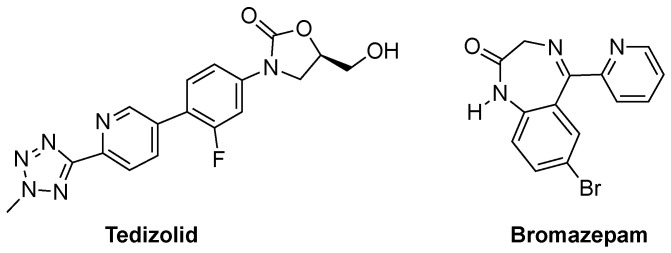
Drugs with a pyridinimine motif.

Similarly, pyridinimines are very important motifs in several drugs. They are present, for example, in Tedizolid [30], an antibiotic of the oxazolidinone class, and in Bromazepam, which is an anti-anxiety agent (Scheme 2) [31].

## 2. Results

### 2.1. N-H-Unsubstituted Pyrrolimines and Pyridinimines

#### 2.1.1. Selected Imines

Considering only N-unsubstituted imines directly linked to the aromatic ring, we failed in our attempts to find in the scientific literature such compounds with a pyrrole substituent for aldimines as well as for ketimines, including N-alkylpyrrole derivatives; however, as early as 1964, the synthesis of C-alkyl derivatives with a pyridine substituent were reported, and among them were α-methyl-2-, 3-, and 4-pyridinylmethanimines, but without spectroscopic characterization [32]. More recently, the ^1^H and ^13^C NMR spectra and HRMS of the α-methyl-3-pyridinemethanimine have been reported [33]. Pyridinylaldimines were also postulated as intermediates [34].

**Scheme 3:**
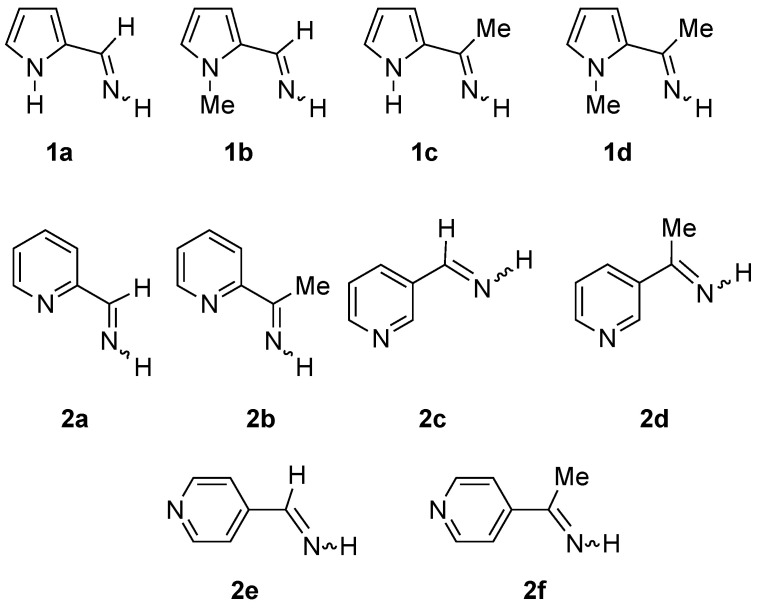
Target imines **1a**–**1d** and **2a**–**2f**.

We selected 10 imines **1a**–**1d** and **2a**–**2f** (Scheme 3). We then considered their synthesis by dehydrocyanation of the corresponding α-aminonitriles and by a retro-ene reaction from N-allylic derivatives. Both precursors will be presented below.

#### 2.1.2. Synthesis of the α-Aminonitriles **3a**–**3d** and **4a**–**4f**

**Scheme 4:**
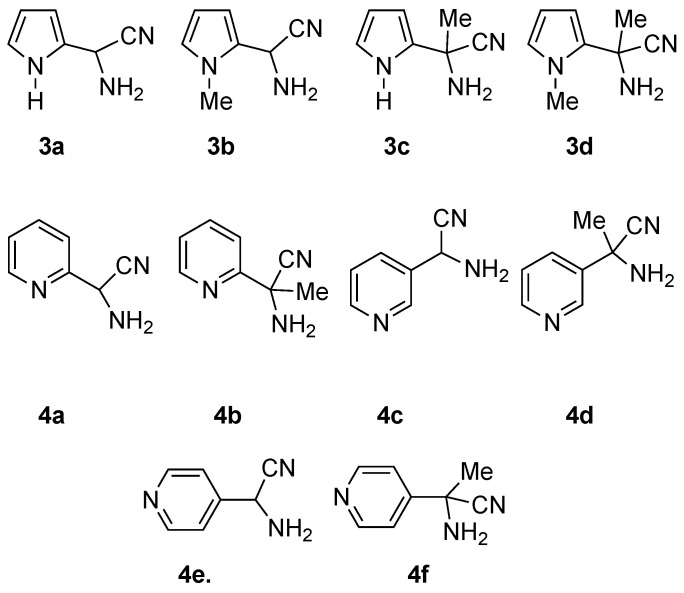
α-aminonitriles **3a**–**3d** and **4a**–**4f**, potential precursors of imines **1a**–**1d** and **2a**–**2f**.

We thus first investigated the synthesis of the ten α-aminonitriles **3a**–**3d** and **4a**–**4f**, potential precursors of these imines (Scheme 4).

The synthesis of compounds **3b**–**3d** has never been reported, while a patent [35] described compound **3a**. The α-aminonitriles **3a** and **3b** were easily synthesized in a Strecker reaction, but their purification was problematic. Distillation in vacuo as well as chromatography on silica gel failed to increase the purity. Attempts to synthesize the two methyl-derivatives **3c** and **3d** were unsuccessful even when the reaction was carried out for a longer time. These results show a significant difference with the derivatives where the aromatic ring is a furan or a thiophene [17,18]. This could be attributed to the presence of the second nitrogen in the molecule (that of the pyrrole substituent) which could interact with the imine intermediate.

The pyrrole ring is π-excessive and the N electron lone pair is part of the aromaticity, while the pyridine ring is π-deficient or not and its N electron lone pair can interact with electrophiles. Different behaviors are therefore possible for each family of compounds.

**Scheme 5:**
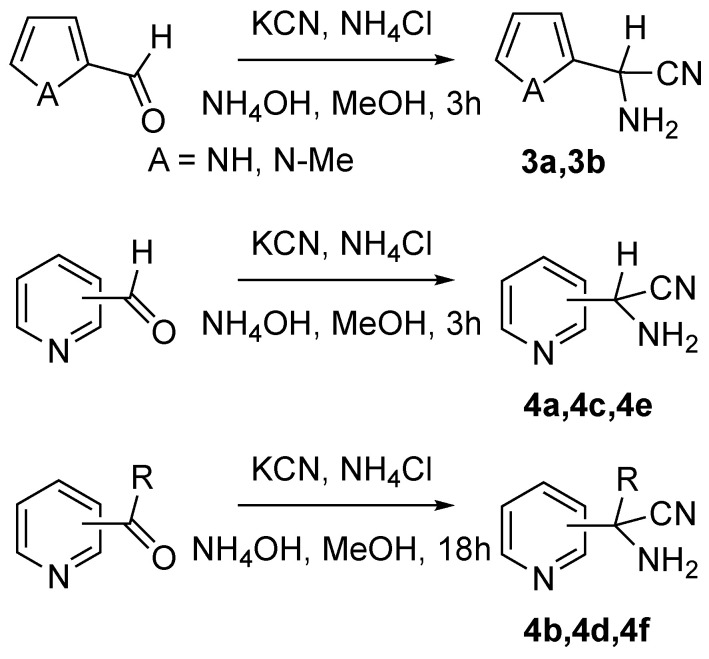
Synthesis of α-aminonitriles **3a**, **3b**, and **4a**–**4f**.

Thus, the synthesis of α-aminonitriles **4a**–**4e** have been reported [36,37,38,39,40], and compounds **4a**–**4f** were readily synthesized in a Strecker reaction, although the methyl derivatives required a longer reaction time. The new compound **4f**, obtained in a 90% yield, was easily characterized by ^1^H and ^13^C NMR spectroscopy, infrared spectroscopy and mass spectrometry, and a comparison of its NMR spectra with those of the non-methylated compound **4e** (Scheme 5, Table 1).

#### 2.1.3. Synthesis of the N-Allylamines **5a**–**5d** and **6a**–**6f**

**Scheme 6:**
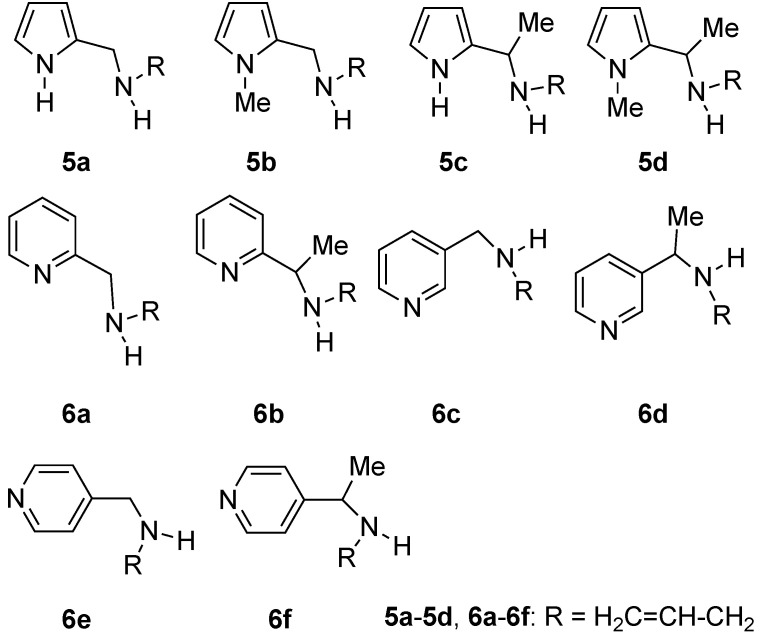
N-allylamines **5a**–**5d** and **6a**–**6f**.

Similarly, the ten N-allylamines **5a**–**5d** and **6a**–**6f** are potential precursors of these imines **1a**–**d** and **2a**–**f** (Scheme 6).

**Scheme 7:**
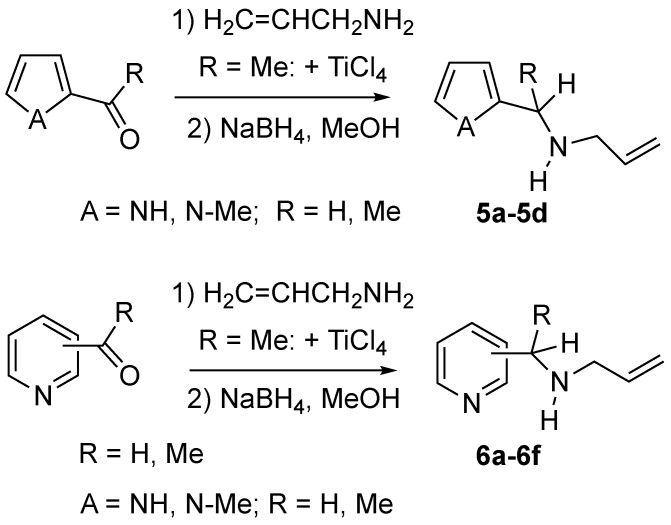
Synthesis of Allylamines **5a**–**5d** and **6a**–**6f**.

For both families of compounds, the synthesis of the N-allylamines **5a**, **5b**, **6a**, **6c**, and **6e** [41,42,43,44,45] has already been reported, but not that of the C-methylated derivatives **5c**, **5d**, **6b**, **6d**, and **6f**. We synthesized all of them by the addition of allylamine to the corresponding aldehyde or ketone, followed by the reduction with NaBH_4_ of the formed imines to the expected amines [44,46,47]. The N-allylamines were obtained in good yields (89–93%) and characterized by ^1^H and ^13^C NMR spectroscopy and a comparison of their spectra with those reported in the literature for the known compounds. For the others, infrared spectra and high-resolution mass spectra (HRMS) were added to confirm their structure (see Supplementary Materials) (Scheme 7, Table 2)**.**

#### 2.1.4. Synthesis of and Attempts to Synthesize the Imines **1a**–**1d** and **2a**–**2f**

The vaporization of α-aminonitriles on hot powdered potassium hydroxide (90 °C) (route A) and retro-ene reactions by thermolysis at 800 °C of N-allylic derivatives (route B), both under vacuum, were therefore used with the aim of synthesizing imines **1a**–**1d** and **2a**–**2f**.

The problem of the vaporization of α-aminonitrile precursors encountered during the synthesis of furan and mainly thiophene derivatives becomes more important for α-aminonitriles substituted by a pyrrole or pyridine group. We considered this difficulty logical knowing the boiling points of pyridine, pyrrole, and 1-methylpyrrole, which are, respectively, 115, 129, and 112 °C higher than that of furane (31 °C) and thiophene (84 °C), as already reported above. It should also be remembered that compounds with a hydrogen on the nitrogen of the pyrrole are much more reactive than the others, with this hydrogen being able to lead to undesirable reactions.

**Scheme 8:**
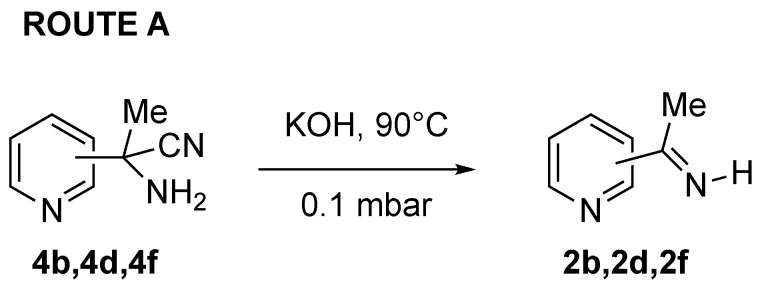
Dehydrocyanation of α-aminonitriles **4b**, **4d**, and **4f**.

From the α-aminonitriles **4b**, **4d**, and **4f**, the pyridinimines **2b**, **2d**, and **2f** were obtained in good yields of 75, 72, and 70%, respectively, and with a good purity when the crude product was condensed in a cold trap cooled at −50 °C before revaporization. Similar experiments with α-aminonitriles **3a**, **3b**, **4a**, **4c**, and **4e** (2.0 mmol) were unsuccessful (Scheme 8, Table 3). The attempt to synthesize **1c** and **1d** by this route was not carried out because the precursors **3c** and **3d** were not prepared.

Overall, the synthesis of pyrrolaldimines and pyridinaldimines by the dehydrocyanation reaction is not satisfactory. Only ketimines with a pyridine ring as a substituent have been obtained in good yields and purity. A much higher instability of aldimines—compounds generally more kinetically unstable than the corresponding ketimines—on hot powdered KOH is probably a parameter of these reactions, but a higher instability on heating of the precursors of aldimines than those of ketimines can also be considered. Therefore, in order to have more efficient syntheses, we proceeded to their preparation by route B, a retro-ene reaction from N-allylamines **5a**–**5d** and **6a**–**6f** by thermolysis at 800 °C under flash vacuum thermolysis conditions.

**Scheme 9:**
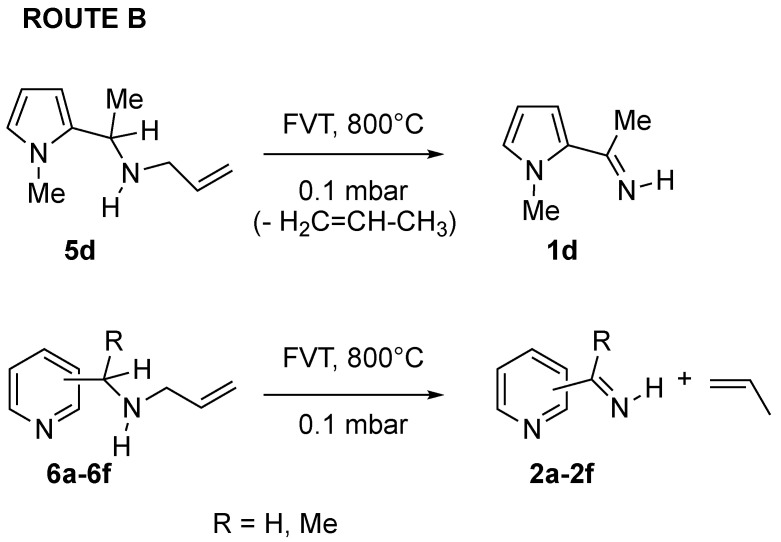
Synthesis imines **1d** and **2a**–**2f** by a retro-ene reaction.

By FVT, N-methyl-α-methyl-2-pyrrolemethanimine **1d** and the three pyridineketimines **2b**, **2d,** and **2f** were synthesized with good yields ranging from 77 to 80%. Moreover, this approach also allows the synthesis of the first pyridinaldimines: pyridinemethanimines **2c** and **2e** were obtained with yields of 76 and 80% in the presence of propene, and characterized by ^1^H and ^13^C NMR spectroscopy. Imine **2a** was obtained with a yield of 23% in the presence of impurities whose major products are pyridine and propene. The purification of **2a**, **2c** and **2e** by condensation–revaporization was not successful because they decomposed at a temperature below the vaporization temperature under vacuum (0.1 mbar). On the other hand, the by-products are too abundant with, at best, traces of pyrrolimines **1a**–**1c** to consider that they have been synthesized (Scheme 9, Table 4).

The half-life of pyrrolimine **1d** (5% in CDCl_3_ and under dry nitrogen) is about 2 days at room temperature, and no decomposition was observed after 2 days for ketimines **2b**, **2d**, and **2f** in such a dilution. Under the same conditions, the half-life of aldimines **2a**, **2c**, and **2e** was about 10 min. Unidentified decomposition products containing pyridine rings were obtained.

**Scheme 10:**
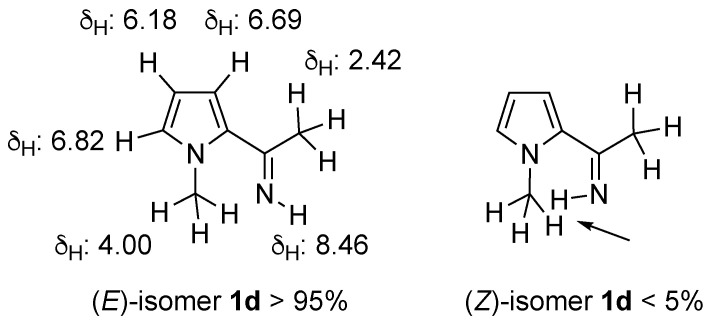
(*E*)- and (*Z*)-N-methyl α-methyl-2-pyrrolemethanimines **1d** and ^1^H NMR data of **(*E*)-1d**.

The ^1^H NMR spectrum of imine **1d** shows a 19:1 ratio between the two stereoisomers. By NOESY analysis, the main stereoisomer was assigned to the (*E*) isomer, an expected result given the steric hindrance in the (*Z*) isomer (see Supplementary Materials and Scheme 10). The N-H signal of (*E*)-**1d** and those corresponding to the hydrogens of the pyrrole ring were observed at δ 8.46 ppm and between 6 and 7 ppm, respectively, and the hydrogens of the methyl groups at δ 4.00 and 2.42 ppm. In ^13^C NMR spectroscopy, the carbon of the imine was observed at δ 169.7 ppm, the ring carbons between 105 and 130 ppm, and the methyl groups at δ 39.0 and 29.0 ppm.

**Scheme 11:**
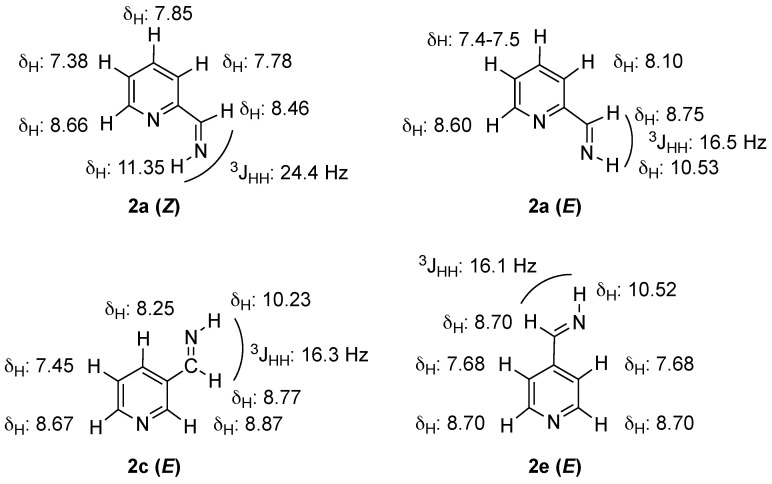
^1^H NMR data of pyridinaldimines **(*Z*)-** and **(*E*)-2a**, **(*E*)-2c**, and **(*E*)-2e**.

The (*E*)- and (*Z*)-pyridinaldimines **2a**, **2c**, and **2e** were characterized by NMR spectroscopy: on the ^1^H NMR spectrum, the signal of the proton on the nitrogen is observed around 9.5 ppm except for the (*Z*)-isomer of **2a** at 11.35 ppm; that on the carbon appears around 8.5 ppm. Coupling constants of about 25 and 16 Hz were easily attributed to the (*Z*)- and (*E*)-isomer, respectively. The chemical shifts in imines on the ^13^C NMR spectra appear between 167 and 169 ppm. For, the cycle, the ^1^H and ^13^C chemical shifts are quite similar to those of substituted aldimines [43,44,45] (Scheme 11).

### 2.2. Reactions of Transimination

**Scheme 12:**
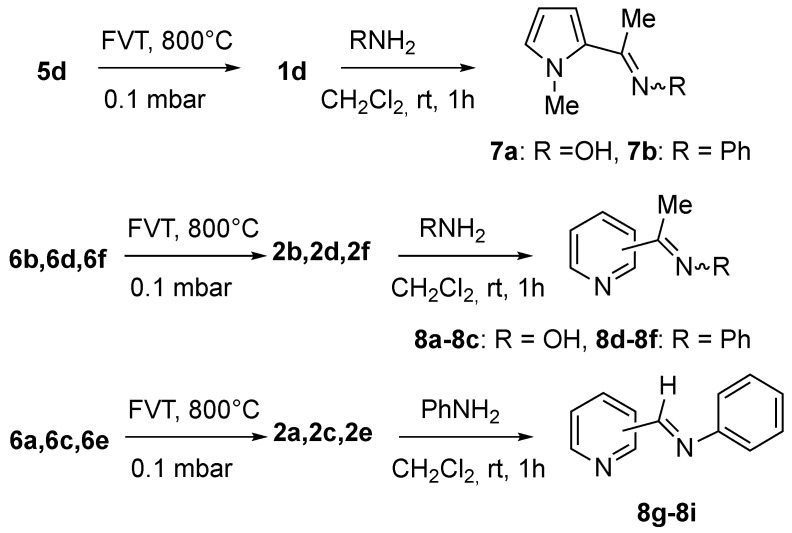
Transimination reaction with imines **1d** and **2a**–**2f**.

**Scheme 13:**
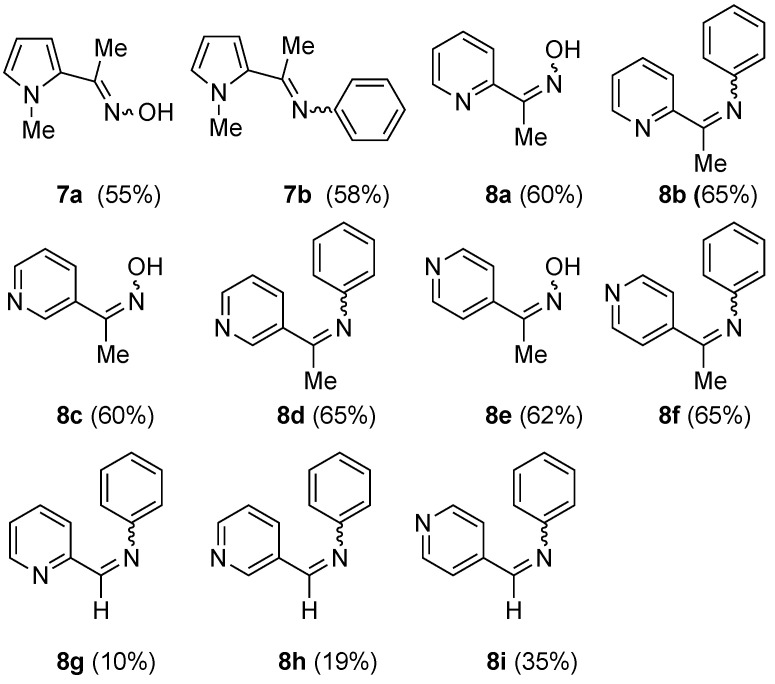
Transimination products with the yield of the two-step reaction.

Since pyrrolimine **1d** and pyridine-ketimines **2b**, **2d**, and **2f** are relatively stable kinetically, transimination should proceed readily unless the ring nitrogen interferes in the reaction. In a two-step reaction from N-allylamines, we reacted hydroxylamine and aniline with pyrrole-ketimine **1d** and the three pyridine-ketimines (**2b**, **2d**, **2f**) to form room-temperature stable N-substituted imines **7a**, **7b**, and **8a**–**8f** (Scheme 12). The aromatic ring nitrogen does not appear to play any role in this reaction. Eight compounds were thus obtained with good yields ranging from 55 to 65% from the amine (Scheme 13).

Pyridinimines **2c** and **2e** with propene and the crude mixture containing imine **2a** were involved in a transamination reaction at the 0.5 mmol scale with aniline as amine. N-phenylimines **8g**–**8i** were obtained in overall yields of 10 (**2a**), 19 (**2c**), and 35% (**2e**) from amines **6a**, **6c**, and **6e**.

All these N-substituted imines were analyzed by ^1^H and ^13^C NMR spectroscopy, and the results are in agreement with those reported in the literature [47,48,49,50,51,52,53,54,55] and also, for **8g**–**8i**, by comparison with authentic samples [54].

### 2.3. Synthesis of Complexed Imines

The complexation of imines by the reaction of an imine with triethylborane has never been described, but N-H complexed aldimines can be obtained by reaction of the corresponding nitrile with superhydride followed by addition of methanol [56]. This approach facilitated an efficient synthesis of complexed aryl [56], furan- [17], and thiophenimines [18] but proved much less efficient with alkylnitriles [56]. In the case of complexed N-H pyrrolaldimines and pyridinaldimines, it was interesting to know the role played by the aromatic ring and in particular by the nitrogen atom during such a synthesis.

**Scheme 14:**
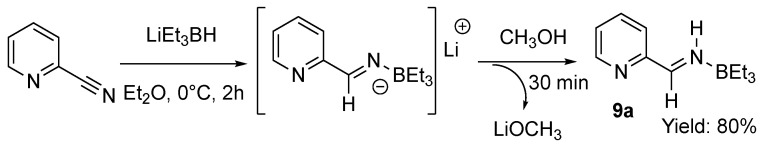
Synthesis of 2-pyridinimine-triethylborane complex **9a**.

Our attempts to synthesize a complexed pyrrolimine from 2-cyanopyrrole were unsuccessful even using an excess of superhydride. However, this approach was found to be very efficient to prepare the 2-pyridinimine-triethylborane complex **9a** from pyridine-2-carbonitrile (Scheme 14). This complex was obtained in 80% yield and characterized by ^1^H, ^13^C, and ^11^B NMR spectroscopy. Surprisingly, the same reaction with pyridine-3-carbonitrile and pyridine-4-carbonitrile gave a complex mixture of products. Unambiguously, the presence of the nitrogen atom on the pyrrole and pyridine ring considerably influences the reactivity of the corresponding nitriles with superhydride while such reactions were really easy with aryl, furan, or thiophene derivatives.

Only the (E) isomer was observed by ^1^H NMR spectroscopy for compound **9a**. The signal of the proton carried by the nitrogen atom is observed as a broad doublet at 10.9 ppm with coupling constants ^3^J = 20.7 Hz. The proton carried by the imine carbon is observed as a doublet at 8.77 ppm.

## 3. Discussion

Pyrrolimine **1d** is a ketimine and an N-methylpyrrole. To our knowledge, compound **1d** is the first N-H pyrrolimine characterized and isolated to date. The synthesis of N-H pyrrolaldimines, and N-H pyrrolketimines N-unsubstituted on the nitrogen of the pyrrole ring therefore remains a challenge that will probably require an approach other than the dehydrocyanation or retro-ene reactions of the corresponding precursors. Compound **1d** was characterized by ^1^H and ^13^C NMR spectroscopy. It is observed mainly as the (*E*) isomer, with steric hindrance appearing evident for the (*Z*) isomer between the hydrogen of the N-H and the methyl group on the other nitrogen (Scheme 10). Compared to the corresponding α-methyl-2-furanmethanimine [17] and α-methyl-2-thiophenemethanimine [18], we observe a chemical shift upfield (δ 8.76 ppm) for the proton signal on the nitrogen atom that could be attributed to a donor effect of the methyl group of the pyrrole moiety. The ^13^C chemical shift in the imine carbon is similar for these three compounds, which otherwise exhibit comparable kinetic stability.

The dehydrocyanation of α-aminonitriles over hot KOH (90 °C) and under vacuum as well as the vacuum thermolysis of N-allylamines provide two routes to synthesize the three N-H pyridineketimines **2b**, **2d**, and **2f** and obtain them in pure form by condensation and revaporization. In addition, this second approach via a retro-ene reaction allows the synthesis of the first pyridinaldimines, the 3-pyridinemethanimine **2c** and 4-pyridinemethanimine **2e**, which were thus obtained with a yield of 76 and 80%, respectively, in the presence of slightly more than one equivalent of propene, while 2-pyridinemethanimine **2a** was obtained in the presence of several impurities and with a lower yield (23%).

**Scheme 15:**
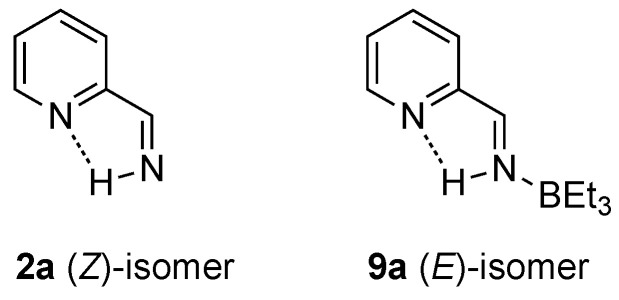
Proposed H-bond in compounds **2a** and **9a**.

These (*Z*)-isomers of 2-pyridinimines **2a** and **2b** exhibit a peculiar property with a chemical shift at downfield for the hydrogen linked to the nitrogen. This could be attributed to a hydrogen bond with the nitrogen atom of the cycle. Such a bond has already been proposed for 2-furanemethanimine [17] but not for 2-thiophenemethanimine [18]. This leads, for **2a**–**2f,** to huge differences in the *Z*/*E* ratio, with the (*E*) isomer being more abundant for the 3-pyridine and 4-pyridine derivatives (**2c**: *Z*/*E* = 1/8; **2e**: *Z*/*E* = 1/17) and not for the 2-pyridine derivative (**2a**: *Z*/*E* = 5/3). Similarly, the usual chemical shift upfield due to complexation [17,18,55,57] was not observed for the (*E*)-2-pyridinemethanimine-triethylborane complex **9a** and could also be attributed to hydrogen bonding (Scheme 15).

Such free pyridinaldimines are kinetically unstable with a very short half-life evaluated to 10 min at room temperature under nitrogen and diluted in a solvent (5% in CD_2_Cl_2_, CDCl_3_, CH_3_CN), showing a higher reactivity than the corresponding furan-, thiophen- and phenyl-aldimines.

The preparation of N-H pyrrolaldimines and pyridinaldimines is therefore much more difficult than for the corresponding furan-, thiophen-, and arylaldimines [17,18,33]. We are clearly, and even more than for thiophenimines, at the limit of an approach to generating the products in the gas phase before condensation on a cold finger. Based on numerous scientific publications on the chemistry of arylaldimines, the chemistry of these pyridinaldimines is currently in progress in our laboratory as well as the search for an approach to synthesize pyrrolaldimines. It should be noted that N-H cyclopentadienimines are also still unknown to date.

## 4. Materials and Methods

I. Route A: General procedure for the synthesis of imines 2b, 2d, and 2f by dehydrocyanation**.**

A reactor (ϕ = 2.0 cm, L = 40 cm) half-filled with KOH powder (39 g, 0.7 mol) was placed in a vacuum line (0.1 mbar) between a reagent inlet on one side and a solvent inlet, a nitrogen gas inlet, and a cold finger on the other. The KOH was heated to 90 °C by a circulating bath, and α-aminonitriles **4b**, **4d**, and **4f** (2.0 mmol) was slowly vaporized into the reactor. The imine formed was condensed in a U-tube immersed in a cold bath cooled to −50 °C to remove propene. At the end of the reaction, the U-tube was allowed to warm to room temperature, and the imine was condensed on the cold finger cooled with liquid nitrogen. A solvent was added at this step. At the end of the addition, the pump was disconnected, the assembly was filled with dry nitrogen, and the liquid nitrogen in the cold finger was expelled with compressed air. The imine and solvent flowed rapidly upon fusion in the NMR tube or in the flask fitted to the bottom of the cold finger and immersed in a liquid nitrogen bath. Pyridinimines **2b**, **2d**, and **2f** were obtained in good purity and with a good yield by this approach. Similar experiments with α-aminonitriles **3a**, **3b**, **4a**, **4c**, and **4e** (2.0 mmol), even without intermediate trapping in the U-tube, failed.

II. Route B: General procedure for the synthesis of imines 1d and 2a–2f by retro-ene reaction.

Compounds **6a**, **6c**, and **6e** (0.5 mmol) were vaporized under vacuum (0.1 mbar) in a quartz tube (L = 35 cm, ϕ = 25 mm) heated in an oven to 800 °C, and the products were directly condensed on a cold finger cooled with liquid nitrogen. A solvent was added at this step. It should be noted that heating to about 50 °C with a heat gun of the connection between the oven and the cold finger is necessary to avoid condensation and decomposition of imines **2a**, **2c**, and **2e** on the wall. A solvent was added, and the next step was identical to that reported above. Pyridinimines **2c** and **2e** were obtained in the presence of slightly more than one equivalent of propene. Several impurities (mainly pyridine and propene) were present with imine **2a**. Yields were determined by ^1^H NMR spectroscopy with an internal reference.

Compounds **5d**, **6b**, **6d**, and **6f** (2.0 mmol) were vaporized under vacuum in the same way and the products were trapped in a U-tube immersed in a cold bath cooled at −50 °C to remove propene. The next step was identical to the one reported above. Imines **1d**, **2b**, **2d**, and **2f** were obtained in good purity and with a good yield by this approach.

Vaporizing **5a**–**5c** with the same approach led to a product mixture too complex to consider that imines **1a**–**1c** were synthesized.

III. Spectroscopic data of imines **1d** and **2a**–**2f**. (Mass spectrometry (HRMS) was not performed for known compounds **2b**, **2d**, and **2e** and was not obtained for kinetically unstable compounds **2a**, **2c**, and **2e.**)


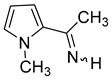


**N-Methyl-α-methyl-2-pyrrolemethanimine** (**1d**). Yield: 80% (195 mg, 1.6 mmol, **route B**). E/Z = 19/1. (*E*) **^1^H NMR** (400 MHz, CDCl_3_) δ 8.76 (s, 1H, NH), 6.82 (m, 1H), 6.69 (m, 1H), 6.18 (m, 1H), 4.00 (s, 3H, N-CH_3_), 2.42 (s, 3H, CH_3_). **^13^C{^1^H} NMR** (100 MHz, CDCl_3_) δ 168.2, 130.4, 128.9, 115.5, 107.1, 38.9, 29.1. **IR** (KBr, film, cm^−1^): 3333 (m), 1609 (m, ν_C=N_), 1432 (s), 1289 (m). **HRMS** (ASAP): [M + H]^+^ calculated for C_7_H_11_N_2_^+^ *m/z* 123.09167, found *m/z* 123.0916.


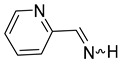


**2-Pyridinemethanimine** (**2a**). Yield: 23% (0.12 mmol, **route B**). (τ_1/2_ (5% (crude) in CDCl_3_ ≈ 7 min). Z/E = 5/3. (*Z*) **^1^H NMR** (400 MHz, CD_2_Cl_2_, 203 K) δ 11.35 (d, *^3^J* = 24.4 Hz, 1H, NH), 8.66 (m, 1H), 8.46 (d, *^3^J* = 24.4 Hz, 1H, CH=N), 7.85 (m, 1H) 7.78 (m, 1H), 7.38 (m, 2H). **^13^C{^1^H} NMR** (100 MHz, CD_2_Cl_2_, 203 K) δ 167.2, 155.1, 149.8, 138.2, 126.4, 121.6 (*E*) **^1^H NMR** (400 MHz, CD_2_Cl_2_, 203 K) δ 10.53 (d, *^3^J* = 16.5 Hz, 1H, NH), 8.75 (d, *^3^J* = 16.5 Hz, 1H, CH=N), 8.60 (m, 1H), 8.10 (m, 1H), 7.5–7.4 (m, 2H). **^13^C{^1^H} NMR** (100 MHz, CD_2_Cl_2_, 203 K) δ 171.6, 155.1, 149.6, 137.9, 124.8, 120.0.


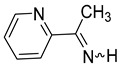


**α-Methyl-2-pyridinemethanimine** (**2b**) [32]. Yield: 75% (180 mg, 1.50 mmol, **route A**), 80% (192 mg, 1.60 mmol, **route B**). *Z*/*E* = 3/1. (*Z*)**^1^H NMR** (400 MHz, CD_2_Cl_2_, 203 K) δ 10.98 (s, 1H, NH), 8.42 (m, 1H), 7.60 (m, 1H), 7.40 (m, 1H), 7.14 (m, 1H), 2.29 (s, 3H, CH_3_). **^13^C{^1^H} NMR** (100 MHz, CD_2_Cl_2_, 203 K) δ 171.7, 150.1, 149.3, 137.7, 125.1, 120.8, 22.2. (*E*) **^1^H NMR** (400 MHz, CD_2_Cl_2_, 203 K) δ 9.62 (s, 1H, NH), 8.42 (m, 1H), 8.00 (m, 1H), 7.57 (m, 1H), 7.14 (m, 1H), 2.50 (s, 3H, CH_3_). **^13^C{^1^H} NMR** (100 Hz, CD_2_Cl_2_, 203 K) δ 175.9, 155.5, 148.4, 136.6, 125.1, 121.0, 26.0. **IR** (KBr, film, ν cm^−1^): 3396 (s), 1645 (m, ν_C=N_), 1459 (m).


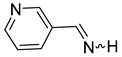


**3-Pyridinemethanimine** (**2c**). Yield: 76% (40 mg, 0.38 mmol, **route B**). (τ_1/2_ (5% in CDCl_3_ ≈ 10 min). E/Z = 8/1. (*E*) **^1^H NMR** (400 MHz, CD_2_Cl_2_, 198 K) δ 10.23 (d, *^3^J* = 16.3 Hz, 1H, NH), 8.87 (s, 1H), 8.77 (d, 1H, *^3^J* = 16.3 Hz, CH=N), 8.67 (s, 1H), 8.25 (m, 1H), 7.45 (m, 1H). **^13^C{^1^H} NMR** (100 MHz, CD_2_Cl_2_, 198 K) δ 168.1, 152.4, 150.6, 134.2, 132.0, 124.2. (*Z*) **^1^H NMR** (400 MHz, CD_2_Cl_2_, 198 K) δ 10.52 (d, *^3^J* = 25.2 Hz, 1H, NH), 8.88 (s, 1H), 8.78 (d, *^3^J* = 25 Hz, 1H, CH=N); the 3 other signals were not unambiguously identified.


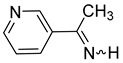


**α-Methyl-3-pyridinemethanimine** (**2d**) [32,33]**.** Yield: 72% (173 mg, 1.44 mmol, **route A**), 77% (185 mg, 1.54 mmol, **route B**). E/Z = 4/1. (*E*)**^1^H NMR** (400 MHz, CDCl_3_, 223 K) δ 9.54 (s, 1H, NH), 9.06 (m, 1H), 8.70 (m, 1H), 8.30 (m, 1H), 7.42 (m, 1H), 2.53 (s, 3H, CH_3_). **^13^C{^1^H} NMR** (100 MHz, CDCl_3_, 223 K) δ 173.1, 151.8, 148.5, 134.6, 133.4, 123.8, 27.4. (*Z*) **^1^H NMR** (400 MHz, CDCl_3_, 223 K) δ 9.80 (s, 1H, NH), 8.82 (m, 1H), 8.61 (m, 1H), 7.89 (m, 1H), 7.28 (m, 1H), 2.53 (s, 3H, CH_3_). **IR** (KBr, film, ν cm^−1^): 3411 (s), 1625 (m, ν_C=N_), 1409 (f).


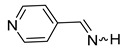


**4-Pyridinemethanimine** (**2e**). Yield: 80% (42 mg, 0.4 mmol, **route B**). (τ_1/2_ (5% in CDCl_3_ ≈ 10 min). E/Z = 17/1. (*E*)**^1^H NMR** (400 MHz, CD_2_Cl_2_, 198 K) δ 10.52 (d, *^3^J* = 16.1 Hz, 1H, NH), 8.71 (m, 2H), 8.70 (d, *^3^J* = 16.1 Hz, 1H, CH=N), 7.68 (m, 2H). **^13^C{^1^H} NMR** (100 Hz, CD_2_Cl_2_, 198 K) δ 169.0, 150.7, 143.0, 121.8. (*Z*) **^1^H NMR** (400 MHz, CD_2_Cl_2_, 198 K) δ 10.95 (d, *^3^J* = 25.4 Hz, 1H, NH), 8.80 (d, *^3^J* = 25.4 Hz, 1H, CH=N), 8.48 (m, 2H), 7.48 (m, 2H).


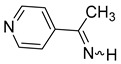


**α-Methyl-4-pyridinemethanimine** (**2f**) [32]. Yield: 70% (168 mg, 1.40 mmol, **route A**), 80% (192 mg, 1.60 mmol, **route B**). E/Z = 4/1. (*E*)**^1^H NMR** (400 MHz, CDCl_3_, 223 K) δ 9.78 (s, 1H, NH), 8.65 (m, 2H), 7.70 (m, 2H), 2.43 (s, 3H, CH_3_). **^13^C{^1^H} NMR** (100 Hz, CDCl_3_, 223 K) δ 173.6, 150.4, 144.9, 121.2, 27.0. (*Z*) **^1^H NMR** (400 MHz, CDCl_3_, 223 K) δ 9.97 (s, 1H, NH), 8.60 (m, 2H), 7.38 (m, 2H), 2.43 (s, 3H, CH_3_). **^13^C{^1^H} NMR** (100 MHz, CDCl_3_, 223 K) δ 175.1, 150.7, 144.9, 119.9, 24.3. **IR** (KBr, film, ν cm^−1^): 3440 (s), 1643 (m, ν_C=N_), 1404 (f).

IV. Synthesis of pyridinemethanimine-triethylborane complex (**9a**). To a solution of pyridine-2-carbonitrile (0.505 g, 4.85 mmol) in diethyl ether (10 mL), lithium triethylborohydride (1M in THF; 4.85 mL, 4.85 mmol) was added at 0 °C, and the solution was stirred for 2 h at 0 °C. Methanol (155 mg, 4.85 mmol) was added to the reaction mixture and stirred for 30 min. The solvent was then evaporated under reduced pressure and the residue was dissolved in dry pentane (5 mL) and filtered through a Kramer filter. Pyridinemethanimine-triethylborane complex **9a** was then precipitated out by slowly cooling the solution to −80 °C, removing the liquid using a pipette, and drying in vacuo. Only the (*E*) isomer of **9a** was obtained in good yield (80%). It is stable at room temperature. (Mass spectrometry (HRMS) analyses are never obtained for such compounds [17,18,55,57].)


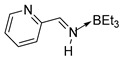


***(E*)-2-pyridinemethanimine-triethylborane complex** (**9a**). Yield: 80% (0.79 g, 3.9 mmol). **^1^H NMR** (400 MHz, CDCl_3_) δ 10.91 (d, *^3^J* = 20.7 Hz, 1H, NH), 8.77 (d, *^3^J* = 4.8 Hz, 1H, CH=N), 8.11, 7.94, 7.61, 7.55 (m, 4H, H-cycle), 0.76 (t, *^3^J* = 7.8 Hz, 9H, 3 CH_3_), 0.36 (d, *J* = 7.9 Hz, 6H, 3 CH_2_). **^13^C{^1^H} NMR** (100 MHz, CDCl_3_) δ 160.7, 150.7, 146.2, 138.0, 127.6, 126.2, 9.7. **^11^B{^1^H} NMR** (128 MHz, CDCl_3_) δ -2.20. **IR** (KBr, film, ν cm^−1^): 3295 (m), 2895 (vs), 1656 (m, ν_C=N_), 1400 (s), 1265 (m).

## Figures and Tables

**Table 1:** Yields of compounds **3a**–**3d** and **4a**–**4f**.

Compound	**3a**	**3b**	**3c**	**3d**	**4a**	**4b**	**4c**	**4d**	**4e**	**4f**
Yield (%)	65	70	0	0	82	90	80	89	90	70

**Table 2:** Yields of compounds **5a**–**5d** and **6a**–**6f**.

Compound	**5a**	**5b**	**5c**	**5d**	**6a**	**6b**	**6c**	**6d**	**6e**	**6f**
Yield (%)	92	92	90	91	93	93	93	90	89	93

**Table 3:** Yields of compounds **1a**–**1b** and **2a**–**2f** by dehydrocyanation of the corresponding α-aminonitriles.

Compound	**1a**	**1b**	**2a**	**2b**	**2c**	**2d**	**2e**	**2f**
Yield (%)	0	0	0	75	0	72	0	70

**Table 4:** Yields of compounds **1a**–**1d** and **2a**–**2f** by thermolysis of the corresponding N-allylamines.

Compound	**1a**	**1b**	**1**c	**1**d	**2a**	**2b**	**2c**	**2d**	**2e**	**2f**
Yield (%)	0	traces?	traces?	80	23	80	76	77	80	80

## Data Availability

Data that support the findings of this study are available from the corresponding authors upon reasonable request.

## Supplementary Materials

The following supporting information can be downloaded at: https://www.mdpi.com/article/10.3390/molecules30061239/s1, General procedure for the synthesis of α-aminonitriles **3a**–**3d** and **4a**–**4f**. General procedure for the synthesis of N-allylamines **5a**–**5d**, **6a**–**6f**, and **5a**, **5b**, **6a**, **6c**, and **6e.** General procedure for the synthesis of N-Allylamines **5c**, **5d**, **6b**, **6d**, and **6f**. Two-dimensional NOESY analysis of (E)-N-methyl-α-methyl-2-pyrrolemethanimine **1d.** General procedure for the transimination with N-H pyrrolaldimines and pyridinimines. ^1^H and ^13^C NMR spectra of compounds **3d**, **4b-4f**, **1d**, **2a**–**2f**, and **9a**.
