# Supplementary material for: Primary Pyrrolimines and Pyridinimines
Source: Molecules. 2025 Mar 10;30(6):1239. doi: 10.3390/molecules30061239 (PMC11944330; doi:10.3390/molecules30061239)
Supplement: Supplementary file 1 [file molecules-30-01239-s001.zip › molecules-3479231-supplementary.pdf]

N-Unsubstituted Pyrrolimines and Pyridinimines

Amavi Kpoezoun,[1,2] Gnon Baba,[2] Jean-Claude Guillemin[1,*]

[1] Univ Rennes, Ecole Nationale Supérieure de Chimie de Rennes, CNRS, ISCR – UMR6226, F-35000 Rennes, France, kpoezoun@gmail.com, jean-claude.guillemin@ensc-rennes.fr

[2] Université de Lomé, Département de chimie, Laboratoire de Chimie Organique et des Substances Naturelles, 01 BP 1515 Lomé, Togo, kpoezoun@gmail.com, gnonbaba@gmail.com,

[*] Correspondence: jean-claude.guillemin@ensc-rennes.fr

SUPPORTING INFORMATION

Table of Contents:

I. General Information S2

II. General procedure for the synthesis of α-aminonitriles **3a-3d**,**4a-4f.** S2

III. General procedure for the synthesis of N-Allylamines **5a-5d**,**6a-6f.** S4

III.1. General procedure for the synthesis of N-Allylamines **5a**,**5b**,**6a**,**6c**,**6e.** S4

III.2. General procedure for the synthesis of N-Allylamines **5c**,**5d**,**6b**,**6d**,**6f.** S4

IV. 2D NOESY analysis of (E)-N-methyl-α-methyl-2-pyrrolemethanimine **1d.** S7

V. General procedure for the transimination with N-H pyrrolaldimines and pyridineketimines S7

VI. References S9

VII. ^{1}H and ^{13}C NMR spectra of compounds **3d**,**4b-4f**,**1d**,**2b-2f**,**9a**. S11

Caution. *Potassium cyanide and probably α-aminonitriles are highly toxic compounds. All reactions and handling should be carried out in a well-ventilated hood. After the Strecker reaction, the aqueous phase should be kept alkaline and was treated with hydrogen peroxide or bleach.*

I. General Information.

NMR spectra were recorded on Bruker Avance 400 MHz spectrometer. Spectra were recorded in deuterochloroform referenced to $CHCl_3$ (δ_H: 7.26 ppm) (δ_C: 77.16 ppm). Chemical shifts (δ) are reported in ppm and coupling constants (J) are reported in Hertz. The following abbreviations are used to describe multiplicity: s-singlet, d-doublet, q-quadruplet, m-multiplet, and br-broad. The IR spectra were recorded on a Nicolet Infra-Red spectrometer, model 320 AVATAR. The sample is deposited on a KBr window. The following abbreviations are used: m-medium, s-strong, vs-very strong. High resolution mass spectra were performed using a time of flight Maxis 4G (Bruker Daltonik Gmbh, Bremen, Germany) in Electrospray positive ionization mode. Ionization mode: ASAP.

2-Pyrrolecarboxaldehyde, N-methyl-2-pyrrolecarboxaldehyde, 2-pyridinecarboxaldehyde, 3-pyridinecarboxaldehyde, 4-pyridinecarboxaldehyde, 2-acetylpyrrole, N-methyl-2-acetylpyrrole, 2-acetylpyridine, 3-acetylpyridine, 4-acetylpyridine, superhydride, allylamine, aniline et hydroxylamine were purchased from the Aldrich, Fluorochem and TCI Europe and used without further purification

II. General procedure for the synthesis of α-aminonitriles (3a)-(3d),(4a)-(4f) [S1].

In a 250 mL three necked flask under nitrogen atmosphere and at room temperature were added NH_4Cl (6.7 g, 0.125 mol), methanol (25 mL), NH_4OH (32% in water, 25 mL) and KCN (7.5 g, 0.115 mol). The aldehyde (0.10 mol) was added dropwise over 5 min with vigorous stirring. The mixture was stirred at room temperature for 3 hours and the organic products were then extracted with dichloromethane (3 x 40mL) and dried over $MgSO_4$. After removal of $MgSO_4$ by filtration, the solvent was evaporated under reduced pressure to give α-aminonitrile **3a**,**3b**,**4a**,**4c**,**4e.** From a ketone, the general procedure was used but 18 h of stirring was necessary to obtain **4b**,**4d**,**4f** in good yield. The compound was used in the next step without further purification. This approach failed to prepare **3c**,**3d**.

HRMS is given only for new compounds **3a**,**3b**,**4b**,**4d**,**4f.**

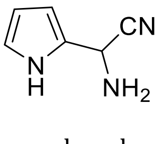

α-Amino-1H-2-pyrrole-2-acetonitrile (3a) [S2]. Yield (crude) from 2-pyrrolecarboxaldehyde: 65% (7.87 g, 65.0 mmol). **^{1}H NMR** (400 MHz, $CDCl_3$) δ 8.80 (s, br, 1H, NH), 6.82 (m, 1H), 6.34 (m, 1H), 6.21 (m, 1H), 4.99 (s, 1H, CH-N), 2.00 (s, br, 2H, NH_2). **^{13}C{^{1}H} NMR** (100 MHz, $CDCl_3$) δ 125.6, 120.3, 119.2, 108.9, 107.4, 41.5. **IR** (KBr, film, ν cm^{-1}): 3370 (vs), 2208 (s, ν_{CN}), 1456 (s), 1263 (m). **HRMS** (ASAP) : $[M+H]^+$ calculated for $C_6H_8N_3^+$ *m/z* 121.0635 , found *m/z* 121.0633.

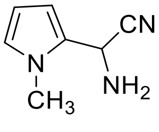

α-Amino-1-methyl-1*H*-pyrrol-2-acetonitrile (3b). Yield (crude) from N-methyl-2-pyrrolecarboxaldehyde: 70% (9.45 g, 70.0 mmol). **^{1}H NMR** (400 MHz, $CDCl_3$) δ 6.68 (m, 1H), 6.35 (m, 1H),

6.11 (m, 1H), 4.92 (s, 1H, CH-N), 3.71 (s, 3H, CH_3), 1.88 (s, 2H, NH_2). **$^{13}C\{^1H\}$ NMR** (100 MHz, $CDCl_3$) δ 126.5, 124.7, 120.4, 108.7, 107.1, 40.8, 34.1. **IR** (KBr, film, ν cm^{-1}): 3366 (s), 2232 (s, ν_{CN}), 1486 (s), 1297 (s). **HRMS** (ASAP): $[M+H]^+$ calculated for $C_7H_{10}N_3^+$ *m/z* 135.0791, found *m/z* 135.0790.

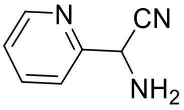

α-Amino-2-pyridinacetonitrile (4a) [S3]**.** Yield from 2-pyridinecarboxaldehyde: 82% (10.9 g , 82.0 mmol). **^1H NMR** (400 MHz, $CDCl_3$) δ 8.59 (m, 1H), 7.72 (m, 1H), 7.48 (m, 1H), 7.20 (m, 1H), 4.99 (s, 1H, CH-CN), 2.70 (s, br, 2H, NH_2). **$^{13}C\{^1H\}$ NMR** (100 MHz, $CDCl_3$) δ 154.9, 149.8, 137.6, 123.9, 121.3, 120.5, 48.8.

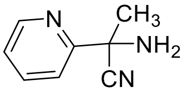

α-Amino-α-methyl-2-pyridinacetonitrile (4b) [S4]**.** Yield from 2-acetylpyridine: 90% (13.2 g, 90.0 mmol). **^1H NMR** (400 MHz, $CDCl_3$) δ 8.60 (m, 1H), 7.74 (m, 1H), 7.63 (m, 1H), 7.26 (m, 1H), 2.36 (s, br, 2H, NH_2), 1.82 (s, 3H, CH_3). **$^{13}C\{^1H\}$ NMR** (100 MHz, $CDCl_3$) δ 159.1, 149.4, 137.4, 123.5, 119.1, 55.1, 29.7. **IR** (KBr, film, ν cm^{-1}): 3364 (vs), 2226 (m, ν_{CN}), 1590 (s). **HRMS** (ASAP): $[M+H]^+$ calculated for $C_8H_{10}N_3^+$ *m/z* 148.0869, found *m/z* 148.0868.

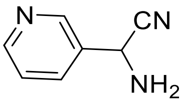

α-Amino-3-pyridinacetonitrile (4c) [S5]**.** Yield from 3-pyridinecarboxaldehyde: 80% (10.6 g, 80.0 mmol). **^1H NMR** (400 MHz, $CDCl_3$) δ 8.74 (m, 1H), 8.58 (m, 1H), 7.89 (m, 1H), 7.36 (m, 1H), 4.97 (s, 1H, CH-CN), 2.14 (s, br, 2H, NH_2). **$^{13}C\{^1H\}$ NMR** (100 MHz, $CDCl_3$) δ 150.1, 148.0, 134.7, 132.3, 123.9, 120.1, 45.2.

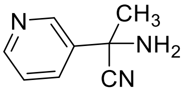

α-Amino-α-methyl-3-pyridinacetonitrile (4d) [S6]**.** Yield from 3-acetylpyridine: 89% (13.1 g, 89.0 mmol). **^1H NMR** (400 MHz, $CDCl_3$) δ 8.95 (m, 1H), 8.63 (m, 1H), 7.98 (m, 1H), 7.35 (m, 1H), 2.19 (s, br, 2H, NH_2), 1.81 (s, 3H, CH_3). **$^{13}C\{^1H\}$ NMR** (100 MHz, $CDCl_3$) δ 150.1, 146.9, 136.9, 133.0, 123.5, 123.1, 52.2, 31.7. **IR** (KBr, film, ν cm^{-1}): 3364 (s), 2224 (m, ν_{CN}), 1598 (m). **HRMS** (ASAP): $[M+H]^+$ calculated for $C_8H_{10}N_3^+$ *m/z* 148.0869, found *m/z* 148.0868.

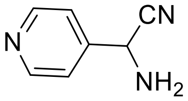

α-Amino-4-pyridinacetonitrile (4e) [S7]**.** Yield from 4-pyridinecarboxaldehyde: 70% (9.31 g, 70.0 mmol). **^1H NMR** (400 MHz, $CDCl_3$) δ 8.50 (m, 2H), 7.38 (m, 2H), 4.93 (s, 1H, CH-CN), 2.14 (s, br, 2H, NH_2). **$^{13}C\{^1H\}$ NMR** (100 MHz, $CDCl_3$) δ 150.3, 145.1, 121.5, 119.7, 46.3.

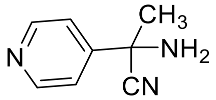

α-Amino-α-methyl-4-pyridinacetonitrile (4f). Yield from 4-acetylpyridine: 90% (13.2 g, 90.0 mmol). **^{1}H NMR** (400 MHz, $CDCl_3$) δ 8.64 (m, 2H), 7.58 (m, 2H), 2.19 (s, br, 2H, NH_2), 1.75 (s, 3H, CH_3). **^{13}C{^{1}H} NMR** (100 MHz, $CDCl_3$) δ 150.5, 150.1, 123.0, 119.9, 53.1, 31.4. **IR** (KBr, film, ν cm^{-1}): 3364 (vs), 2226 (m, ν_{CN}), 1590 (s). **HRMS** (ASAP): $[M+H]^+$ calculated for $C_8H_{10}N_3^+$ *m/z* 148.0869 , found *m/z* 148.0868.

III. General procedure for the synthesis of N-Allylamines (5a)-(5d), (6a)-(6f) [S8,S9]**.**

III.1. General procedure for the synthesis of N-Allylamines 5a,5b,6a,6c,6e.

Allylamine (1.71 g, 30 mmol), dry CH_2Cl_2 (150 mL) and $MgSO_4$ (4.00 g) were stirred, under nitrogen, in a two-necked round bottom flask (500 mL) fitted with a dropping funnel. The aldehyde (25 mmol) diluted in dry CH_2Cl_2 (3 mL) was added dropwise. After 3 h, the mixture was filtered to remove hydrated $MgSO_4$, and concentrated under reduced pressure. The crude products were then dissolved in CH_3OH (50 mL), the mixture was cooled in a water bath and $NaBH_4$ (0.46 g, 12.5 mmol) was gradually added under vigorous stirring. After 5 h, the mixture was diluted with water (100 mL) and extracted with ether (3 x 30 mL). The organic phases were collected, dried with $MgSO_4$ and concentrated under reduced pressure to give N-allylamines **5a**,**5b**,**6a**,**6c**,**6e** in good yields. The crude N-allylamines were sufficiently pure to be used for the thermolysis.

III.2. General procedure for the synthesis of N-Allylamines 5c,5d,6b,6d,6f.

In a 250 mL round flask under nitrogen, diethyl ether (75 mL), ketone (1.9 g, 15 mmol) and allylamine (3.42 g, 60 mmol) were introduced. A solution of $TiCl_4$ (1M) in dichloromethane (7.5 mmol, 0.5 equiv.) was added dropwise at 0 °C over a period of 15 minutes. The reaction mixture was allowed to warm to room temperature and stirred for 2h. The precipitate was filtered through celite and washed with diethyl ether (60 mL). The filtrate was washed with brine (30 mL), dried over $MgSO_4$ and the solvent removed under reduced pressure. The crude products were then dissolved in CH_3OH (50 mL), the mixture was cooled in a water bath and $NaBH_4$ (0.28 g, 7.5 mmol) was gradually added under vigorous stirring. After 5 h, the mixture was diluted with water (60 mL) and extracted with ether (3 x 20 mL). The organic phases were collected, dried with $MgSO_4$ and concentrated under reduced pressure to give N-allylamines **5c**,**5d**,**6b**,**6d**,**6f** in good yield. The crude N-allylamines were pure enough to be used for the following step, the flash vacuum thermolysis.

HRMS is given only for new compounds **5b**,**5d**,**6b**,**6d**,**6f**.

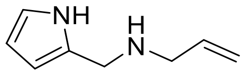

***N*-2-Propen-1-yl-1*H*-pyrrole-2-methanamine (5a)** [S10]**.** Yield from 2-pyrrolecarboxaldehyde: 92% (1.40 g, 10.3 mmol)**. ^{1}H NMR** (400 MHz, $CDCl_3$) δ 9.50 (s, 1H, NH-cycle), 6.76 (m, 1H), 6.11 (m, 1H), 5.95 (ddt, 1H, *J* = 16.3, 10.4, 6.0 Hz, CH=C), 5.25 (ddt, 1H, *J* = 16.3, 1.7, 1.7 Hz, C=C<u>H</u>H),

5.19 (ddt, 1H, *J* = 10.4, 1.7, 1.5 Hz, C=CHH), 3.82 (s, 2H, CH_2), 3.31 (dt, ^3J = 6.1, 1.5 Hz, 2H, CH_2-N), 2.88 (s, br, 1H, NH). **$^{13}C\{^1H\}$ NMR** (100 MHz, $CDCl_3$) δ 136.0, 129.7, 117.9, 116.7, 107.8, 107.0, 51.5, 45.8.

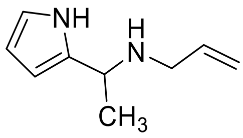

α-Methyl-*N*-2-Propen-1-yl-1*H*-pyrrole-2-methanamine (5b). Yield from 2-acetylpyrrole: 92% (1.53 g, 10.2 mmol)**.** **^1H NMR** (400 MHz, $CDCl_3$) δ 8.71 (s, 1H, NH-cycle), 6.70 (m, 1H), 6.09 (m, 1H), 5.92 (ddt, 1H, *J* = 17.2, 10.3, 6.0 Hz, CH=C), 5.19 (ddt, 1H, *J* = 17.2, 1.7, 1.7 Hz, C=CHH), 5.11 (ddt, 1H, *J* = 10.3, 1.7, 1.5 Hz, C=CHH), 3.96 (q, ^3J = 6.6 Hz, 1H, CH-N), 3.21 (d, 2H, *J* = 6.1, 1.5 Hz, CH_2-N), 1.70 (s, 1H, NH), 1.42 (d, ^3J = 6.6 Hz, 3H). **$^{13}C\{^1H\}$ NMR** (100 MHz, $CDCl_3$) δ 136.9, 135.3, 116.6, 115.8, 107.9, 104.8, 50.7, 49.8, 22.1. **IR** (KBr, film, ν cm^{-1}): 3283 (m), 3080 (m), 2969 (m), 1436 (s), 1325 (s). **HRMS** (ASAP): $[M+H]^+$ calculated for $C_9H_{15}N_2^+$ *m/z* 151.12352 , found *m/z* 151.1235.

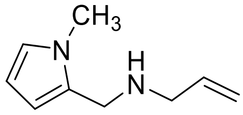

1-Methyl-*N*-2-propen-1-yl-1*H*-pyrrole-2-methanamine (5c) [S11]**.** Yield from N-methyl-2-pyrrolecarboxaldehyde: 90% (1.50 g, 10.0 mmol)**.** **^1H NMR** (400 MHz, $CDCl_3$) δ 6.65 (m, 1H), 6.11 (m, 1H), 6.00 (ddt, 1H, *J* = 16.2, 10.3, 5.9 Hz, CH=C), 5.28 (ddt, 1H, *J* = 16.2, 1.7, 1.7 Hz, C=CHH), 5.18 (ddt, 1H, *J* = 10.3, 1.7, 1.4 Hz, C=CHH), 3.80 (s, 2H, CH_2), 3.71 (s, 3H, CH_3), 3.36 (dt, ^3J = 5.9, 1.6 Hz, 2H, CH_2), 1.39 (s, br, 1H, NH). **$^{13}C\{^1H\}$ NMR** (100 MHz, $CDCl_3$) δ 137.0, 131.2, 122.4, 116.0, 107.9, 106.5, 51.8, 44.8, 33.7.

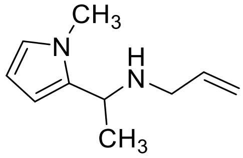

α-Methyl-1-Methyl-*N*-2-propen-1-yl-1*H*-pyrrole-2-methanamine (5d). Yield from N-methyl-2-acetylpyrrole: 91% (1.66 g, 10.1 mmol)**.** **^1H NMR** (400 MHz, $CDCl_3$) δ 6.57 (m, 1H), 6.11 (m, 1H), 5.93 (m, 1H, H-cycle), 6.00 (ddt, 1H, *J* = 17.2, 10.2, 6.0 Hz, CH=C), 5.18 (ddt, 1H, *J* = 17.2, 1.7, 1.7 Hz, C=CHH), 5.08 (ddt, 1H, *J* = 10.2, 1.7, 1.5 Hz, C=CHH), 3.93 (q, ^3J = 6.6 Hz, 1H, CH-N), 3.68 (s, 3H, CH_3-N), 3.22 (d, 2H, *J* = 6.0 Hz CH_2-N), 1.45 (d, ^3J = 6.6 Hz, 3H, CH_3), 1.23 (s, 1H, NH). **$^{13}C\{^1H\}$ NMR** (100 MHz, $CDCl_3$) δ 137.2, 136.1, 122.0, 115.6, 106.4, 105.2, 49.7, 49.2, 34.0, 21.1. **IR** (KBr, film, ν cm^{-1}) : 3306 (m), 3075 (m), 2966 (m), 1444 (m), 1327 (m). **HRMS** (ASAP): $[M+H]^+$ calculated for $C_{10}H_{17}N_2^+$ *m/z* 165.13917, found *m/z* 65.1392.

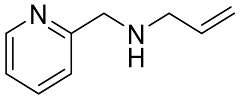

***N*-2-Propen-1-yl-2-pyridinemethanamine (6a)** [S8]**.** Yield from 2-pyridinecarboxaldehyde: 93% (1.53 g, 10.3 mmol)**.** **^1H NMR** (400 MHz, $CDCl_3$) δ 8.56 (m, 1H), 7.64 (m, 1H),

7.30 (m, 1H), 7.16 (m, 1H), 5.95 (ddt, 1H, *J* = 16.2, 10.1, 6.0 Hz, CH=C), 5.23 (ddt, 1H, *J* = 16.2, 1.7, 1.7 Hz, C=C<u>H</u>H), 5.12 (ddt, 1H, *J* = 10.1, 1.7, 1.4 Hz, C=CH<u>H</u>), 3.91 (d, ^3J = 7.1 Hz, 2H, CH_2), 3.32 (d, ^3J = 6.0 Hz, 2H, CH_2), 1.88 (s, br, 1H, NH). **$^{13}C\{^1H\}$ NMR** (100 MHz, $CDCl_3$) δ 159.8, 149.4, 136.0, 122.3, 121.9, 116.1, 54.5, 52.0.

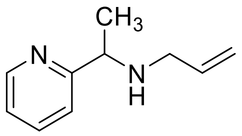

α-Methyl-*N*-2-propen-1-yl-2-pyridinemethanamine (6b). Yield from 2-acetylpyridine: 93% (1.67 g, 10.3 mmol). **^1H NMR** (400 MHz, $CDCl_3$) δ 8.55 (m, 1H), 7.64 (m, 1H), 7.27 (m, 1H), 7.15 (m, 1H), 5.88 (ddt, 1H, *J* = 16.2, 10.8, 6.0 Hz, CH=C), 5.13 (ddt, 1H, *J* = 16.2, 1.7, 1.7 Hz, C=C<u>H</u>H), 5.08 (ddt, 1H, *J* = 10.8, 1.7, 1.4 Hz, C=CH<u>H</u>), 3.89 (q, ^3J = 6.7 Hz, 1H, CH-N), 3.10 (d, 2H, *J* = 5.9 Hz, CH_2-N), 2.16 (s, br, 1H, NH), 1.39 (d, ^3J = 6.7 Hz, 3H, CH_3). **$^{13}C\{^1H\}$ NMR** (100 MHz, $CDCl_3$) δ 164.4, 149.3, 136.8, 136.5, 121.9, 121.3, 115.8, 58.5, 50.3, 22.7. **IR** (KBr, film, ν cm^{-1}) : 3289 (m), 3075 (m), 2973 (m), 1433 (vs), 1338 (m). **HRMS** (ASAP): $[M+H]^+$ calculated for $C_{10}H_{15}N_2^+$ *m/z* 163.12297, found *m/z* 163.1231.

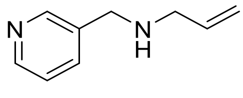

***N*-2-Propen-1-yl-3-pyridinemethanamine (6c).** Yield from 3-pyridinecarboxaldehyde: 90% (1.48 g, 10.0 mmol). **^1H NMR** (400 MHz, $CDCl_3$) δ 8.51 (m, 1H), 8.45 (m, 1H), 7.67 (m, 1H), 7.26 (m, 1H), 5.88 (ddt, 1H, *J* = 17.2, 10.8, 6.0 Hz, CH=C), 5.18 (ddt, 1H, *J* = 17.2, 1.7, 1.7 Hz, C=C<u>H</u>H), 5.12 (ddt, 1H, *J* = 10.8, 1.7, 1.4 Hz, C=CH<u>H</u>), 3.80 (d, ^3J = 6.7 Hz, 2H, CH_2), 3.27 (d, 2H, *J* = 6.0 Hz, CH_2), 1.32 (s, br, 1H, NH). **$^{13}C\{^1H\}$ NMR** (100 MHz, $CDCl_3$) δ 149.8, 148.5, 136.4, 135.8, 135.6, 123.4, 116.3, 51.7, 50.5. **IR** (KBr, film, ν cm^{-1}): 3276 (m), 2929 (m), 2815 (m), 1446 (m), 1360 (m).

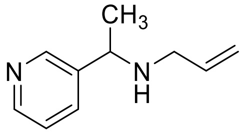

α-Methyl-*N*-2-propen-1-yl-3-pyridinemethanamine (6d). Yield from 3-acetylpyridine : 93% (1.67 g, 10.3 mmol). **^1H NMR** (400 MHz, $CDCl_3$) δ 8.53 (m, 1H), 8.48 (m, 1H), 7.67 (m, 1H), 7.25 (m, 1H), 5.85 (ddt, 1H, *J* = 17.2, 10.4, 6.0 Hz, CH=C), 5.12 (ddt, 1H, *J* = 17.2, 1.6, 1.6 Hz, C=C<u>H</u>H), 5.07 (ddt, 1H, *J* = 10.4, 1.6, 1.5 Hz, C=CH<u>H</u>), 3.84 (q, ^3J = 6.6 Hz, 1H, CH-N), 3.08 (d, 2H, *J* = 6.0 Hz, CH_2-N), 1.37 (s, br, 1H, NH), 1.36 (d, ^3J = 6.6 Hz, 3H, CH_3). **$^{13}C\{^1H\}$ NMR** (100 MHz, $CDCl_3$) δ 148.9, 148.5, 140.6, 136.7, 134.1, 123.5, 116.0, 55.1, 50.1, 24.1. **IR** (KBr, film, ν cm^{-1}): 3281 (m), 3077 (m), 2970 (m), 1424 (s), 1312 (m). **HRMS** (ASAP): $[M+H]^+$ calculated for $C_{10}H_{15}N_2^+$ *m/z* 163.12297, found *m/z* 163.1230.

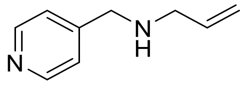

***N*-2-Propen-1-yl-4-pyridinemethanamine (6e).** Yield from 4-pyridinecarboxaldehyde: 89% (1.46 g, 9.88 mmol). **^1H NMR** (400 MHz, $CDCl_3$) δ 8.45 (m, 2H), 7.17 (m, 2H), 5.84 (ddt, 1H, *J* = 16.2, 10.3, 5.9 Hz, CH=C), 5.12 (ddt, 1H, *J* = 16.2, 1.6, 1.6 Hz, C=C<u>H</u>H), 5.04 (ddt, 1H, *J* = 10.3, 1.6, 1.4 Hz, C=CH<u>H</u>), 3.72 (s, 2H, CH_2), 3.19 (m, 2H, *J* = 5.9 Hz, CH_2), 1.67 (s, br, 1H, NH). **$^{13}C\{^1H\}$ NMR** (100

MHz, $CDCl_3$) δ 149.7, 149.4, 136.3, 122.9, 116.3, 51.7, 51.7. **IR** (KBr, film, ν cm^{-1}): 3283 (m), 3072 (m), 2977 (m), 1421 (vs), 1363 (m).

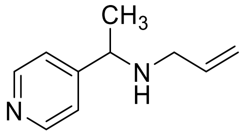

α-Methyl-*N*-2-propen-1-yl-4-pyridinemethanamine (6f). Yield from 4-acetylpyridine: 93% (1.67 g, 10.3 mmol). **^{1}H NMR** (400 MHz, $CDCl_3$) δ 8.46 (m, 2H), 7.20 (m, 2H), 5.80 (ddt, 1H, *J* = 16.2, 10.3, 5.9 Hz, CH=C), 5.07 (ddt, 1H, *J* = 16.2, 1.7, 1.7 Hz, C=C<u>H</u>H), 5.02 (ddt, 1H, *J* = 10.3, 1.7, 1.4 Hz, C=CH<u>H</u>), 3.73 (m, 1H, N-CH), 3.01 (m, 2H, *J* = 6.0 Hz, CH_2-N), 1.30 (s, br, 1H, NH), 1.27 (d, ^3J = 3.3 Hz, 3H). **^{13}C{^{1}H} NMR** (100 MHz, $CDCl_3$) δ 154.6, 149.8, 136.4, 121.9, 116.0, 56.6, 50.1, 23.8. **IR** (KBr, film, ν cm^{-1}): 3270 (m), 3074 (m), 2974 (m), 1414 (vs), 1310 (m). **HRMS** (ASAP): $[M+H]^+$ calculated for $C_{10}H_{15}N_2^+$ *m/z* 163.12297, found *m/z* 163.1230.

IV. 2D NOESY analysis to identify the major stereoisomer of imine (1d).

Imine **1d** has been synthesized as reported above (route B). 2D NOESY analysis was used to determine which of the (*Z*) and (*E*) isomer is the major one. The 2D NOESY analysis shows two correlation spots **E** and **F** between the methyl group (δH_6: 2.42 ppm) linked to the imine function and the nitrogen proton (δH_4: 8.76 ppm) and between the same methyl group (δH_6: 2.42 ppm) and the H_3 proton of the cycle (δH_3: 6.68 ppm). We also observe a correlation spot **I** between the methyl group (δH_5: 4.00 ppm) linked to the nitrogen of the cycle and the H_1 proton of the cycle (δH_1: 6.68 ppm) (Figure S1). The major isomer is thus clearly the (E).

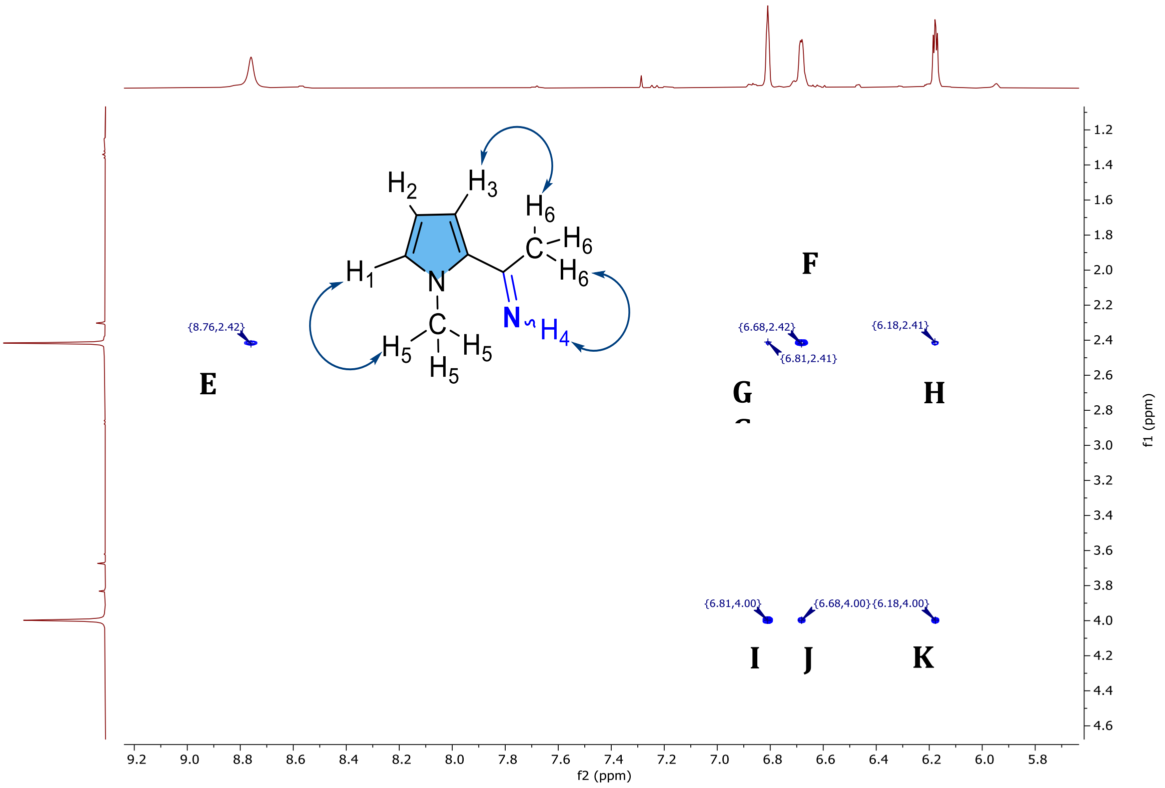

Figure S1. 2D NOESY analysis of (*E*)-N-methyl-α -methyl-2-pyrrolemethanimine **1d**.

Four additional correlation spots are observed between the two methyl groups and the other protons in the cycle. They are represented by the letters **G** (δH_1: 6.81 ppm and δH_6: 2.42 ppm), **H** (δH_2: 6.18 ppm and δH_6: 2.42 ppm), **J** (δH_3: 6.68 ppm and δH_5: 4.00 ppm) and **K** (δH_2: 6.18 ppm and δH_5: 4.00 ppm). They are due to the spatial orientation of the two methyl groups.

V. General procedure for the transimination with N-H pyrrolketimine (1d) and pyridineketimines (2a-2f). The imine **1d**,**2b-2f** was synthesized as reported above starting from allylamine **5d,6b**,**6d**,**6f** (2.0 mmol) or **6a**,**6c**,**6e** (0.5 mmol), and dry dichloromethane (2 mL) was added as solvent. A 50 mL two necked flask was fitted at the bottom of the cold finger and was immersed in a cold bath (-50°C). The imine **1d**,**2b**,**2d**,**2f** and the solvent flowed rapidly as soon as they melted into the flask. The amine (1.1 eq.) was added to the reaction mixture and stirred for 1 h at -50 °C, allowed to warm slowly to room temperature, and stirred for 1 h. The solvent and amine in excess were evaporated under reduced pressure. Overall yields (starting from **5d,6a-6f)** are given below but, on the basis of the yields found for imines **1d**,**2b**,**2d**,**2f**, the yield of the transimination reaction ranges between 75 and 92%. For compounds **7a,7b,8a-8i** the ^{1}H and ^{13}C NMR spectra were identical to those described in the literature [S12-S18]**.**

(E)-1-(1-Methyl-1*H*-pyrrol-2-yl)ethanone oxime 7a [S12]**.** Yield: 55% (0.15 g, 1.1 mmol) from **5d (route B)**.

(*E*)- *N*-[1-(1-Methyl-1*H*-pyrrol-2-yl)ethylidene]benzenamine 7b[S16]**.** Yield: 58% (0.24 g, 1.2 mmol from **5d (route B)**.

***(E)*-*N*-[1-(2-Pyridinyl)ethylene]-oxime (8a)** [S14]**.** Yield: 60% (0.16 g, 1.2 mmol) from **6b (route B)**.

[*N*(*E*)]-*N*-[1-(2-Pyridinyl)ethylidene]benzenamine (8b) [S15]**.** Yield: 65% (0.25 g, 1.3 mmol) from **6b (route B)**.

(1*E*)-1-(3-Pyridinyl)ethanone oxime (8c) [S16]**.** Yield: 60% (0.16 g, 1.2 mmol) from **6d (route B)**.

[*N*(*E*)]-*N*-[1-(3-Pyridinyl)ethylidene]benzenamine (8d) [S17]**.** Yield: 65% (0.25 g, 1.3 mmol) from **6d (route B)**.

(1*E*)-1-(4-Pyridinyl)ethanone oxime (8e) [S18]**.** Yield: 62% (0.17 g, 1.24 mmol) from **6f (route B)**.

***(E)*-*N*-[1-(4-Pyridinyl)ethylene]-benzenamine (8f)** [S17]**.** Yield: 65% (0.25 g, 1.3 mmol) from **6f (route B)**.

Transimination reaction from **2a**,**2c**,**2e** and aniline were performed starting from 0.5 mmol.

[*N*(*E*)]-*N*-[1-(2-Pyridinyl)ethylidene]benzenamine (8g) [S19]**.** Yield: 10% (18 mg, 0.05 mmol) from **6a (route B)**.

[*N*(*E*)]-*N*-[1-(3-Pyridinyl)ethylidene]benzenamine (8h) [S19]**.** Yield: 19% (35 mg, 0.1 mmol) from **6c (route B)**.

***(E)*-*N*-[1-(4-Pyridinyl)ethylene]-benzenamine (8i)** [S19]**.** Yield: 35% (64 mg, 0.18mmol) from **6e (route B)**.

VI. References

S1. Guillemin, J.-C.; Denis, J.-M. Synthese d'imines lineaires non-stabilisees par reactions gaz-solide sous vide(1). *Tetrahedron* **1988**, 44, 4431–4446.

S2. Patent. Preparation and formulation of pyrazolylcarboxamides as agrochemical fungicides, Mitsui Toatsu Chemicals, Inc., Federal Republic of Germany, DE3713774 A1 **1987**-10-29**.**

S3. Chu, G.-H.; Gu, M.; Gerard, B.; Dolle, R. E. Development of a new α-aminonitrile synthesis. *Synth. Comm.* **2004**, 34(24), 4583-4590.

S4. O'Brien, P. M.; Sliskovic, D. R.; Blankley, C. J.; Roth, Bruce. D.; Michael W.; Hamelehle, K. L.; Krause, B. R.; Stanfield, R. L. Inhibitors of Acyl-CoA: Cholesterol O-Acyl Transferase (ACAT) as Hypocholesterolemic Agents. 8. Incorporation of Amide or Amine Functionalities into a Series of Disubstituted Ureas and Carbamates. Effects on ACAT Inhibition in vitro and Efficacy in vivo. *J. Med. Chem.* **1994**, 37(12), 1810-1822**.**

S5. Sauerberg, P.; Olesen, P. H.; Nielsen, S.; Treppendahl, S.; Sheardown, M. J.; Honore, T.; Mitch, C. H.; Ward, J. S.; Pike, A. J.; Novel functional M_1 selective muscarinic agonists. Synthesis and structure-activity relationships of 3-(1,2,5-thiadiazolyl)-1,2,5,6-tetrahydro-1-methylpyridines. *J. Med. Chem.,* **1992**, 35(12), 2274-2283.

S6. Patent. Preparation of peptidomimetics as inhibitors of cysteine proteases for treatment of viral infections including COVID-19, Pardes Biosciences, Inc., World Intellectual Property Organization, WO2023044171 A1 **2023**-03-23

S7. Klosa, J. Uber einige Kondensationen mit Pyridin- und Chinolin-aldehyde, *Archiv der Pharmazie und Berichte der Deutschen Pharmazeutischen Gesellschaft* **1956**, 289, 177-88.

S8. Ghelfi, F.; Parsons, A. F.; Tommasini, D.; Mucci, A. Intramolecular Diels–Alder Cycloaddition of N-Allyl-N-(2-furylmethyl)amides – First Step of a New Route Towards the Synthesis of a Densely Functionalized Pyrrolizidine Ring, *Eur. J. Org. Chem.* **2001**, 10, 1845–1852.

S9. Ghelfi, F.; Parsons, A.F.; Tommasini, D.; Mucci, A. Intramolecular Diels–Alder Cycloaddition of N-Allyl-N-(2-furylmethyl)amides – First Step of a New Route Towards the Synthesis of a Densely Functionalized Pyrrolizidine Ring. *Eur. J. Org. Chem.* **2001**, *10*, 1845–1852. https://doi.org/10.1002/1099-0690(200105)2001:10<1845::AID-EJOC1845>3.0.CO;2-I.

S10. Martínez-Mora, E. I.; Caracas, M. A.; Escalante, C. H.; Espinoza-Hicks, C.; Quiroz-Florentino, H.; Delgado, F.; Tamariz, J. 2-Formylpyrroles as Building Blocks in a Divergent Synthesis of Pyrrolizines. *Synthesis* **2016**, 48, 1055–1068.

S11. Kennemur, J. L.; Kortman, G. D.; Hull, K. L. Rhodium-Catalyzed Regiodivergent Hydrothiolation of Allyl Amines and Imines, *J. Am. Chem. Soc.* **2016,** *138(36),* 11914–11919, https://doi.org/10.1021/jacs.6b07142

S12. Fournari, P. Heterocyclic series. VI. Synthesis of the nitro derivatives of N-methylpyrrole. Application of polarographic analysis to the separation of the nitro isomers. *Bull. Soc. Chim. Fr.* **1963**, 488-491.

S13. Barluenga, J.; Jiménez-Aquino, A.; Aznar, F.; Valdés, C. Modular Synthesis of Indoles from Imines and o-Dihaloarenes or o-Chlorosulfonates by a Pd-Catalyzed Cascade Process*. J. Am. Chem. Soc*. **2009**, 131, 4031–4041.

S14. Chang, Z. Y.; Coates, R. M. Diastereoselectivity of organometallic additions to nitrones bearing stereogenic N-substituents. *J. Org. Chem.* **1990**, 55(11), 3464-3474.

S15. Miller, K. J.; Kitagawa, Terutaka T.; Abu-Omar, M. M. Kinetics and Mechanisms of Methyl Vinyl Ketone Hydroalkoxylation Catalyzed by Palladium(II) Complexes. *Organometallics* **2001**, 20(21), 4403-4412.

S16. LaForge, F. B. Preparation and properties of some new derivatives of pyridine. *J. Am. Chem. Soc.* **1928**, 50, 2477-2483.

S17. Schnell, B. Synthesis and reactions of 4-hydroxy-2(1H)-pyridones with thienyl and pyridyl substituents in position 6 starting with azomethines and malonates. *J. Hetero. Chem.* **1999**, 36(2), 541-548.

S18. LaMattina, J. L.; Suleske, R. T. α-Amino acetals: 2,2-diethoxy-2-(4-pyridyl)ethylamine, *Org. Synth.* **1986**, 64, 19.

S19. Troisi, L., Ronzini, L., Granito, C., De Vitis, L., Pindinelli, E., Stereoselective synthesis and functionalization of 4-heterosubstituted β-lactams, *Tetrahedron* 2006, 62, 1564–1574.

VII. ^{1}H and ^{13}C NMR spectra of compounds (3d),(4b)-(4f),(1d),(2a)-(2f),(9a).

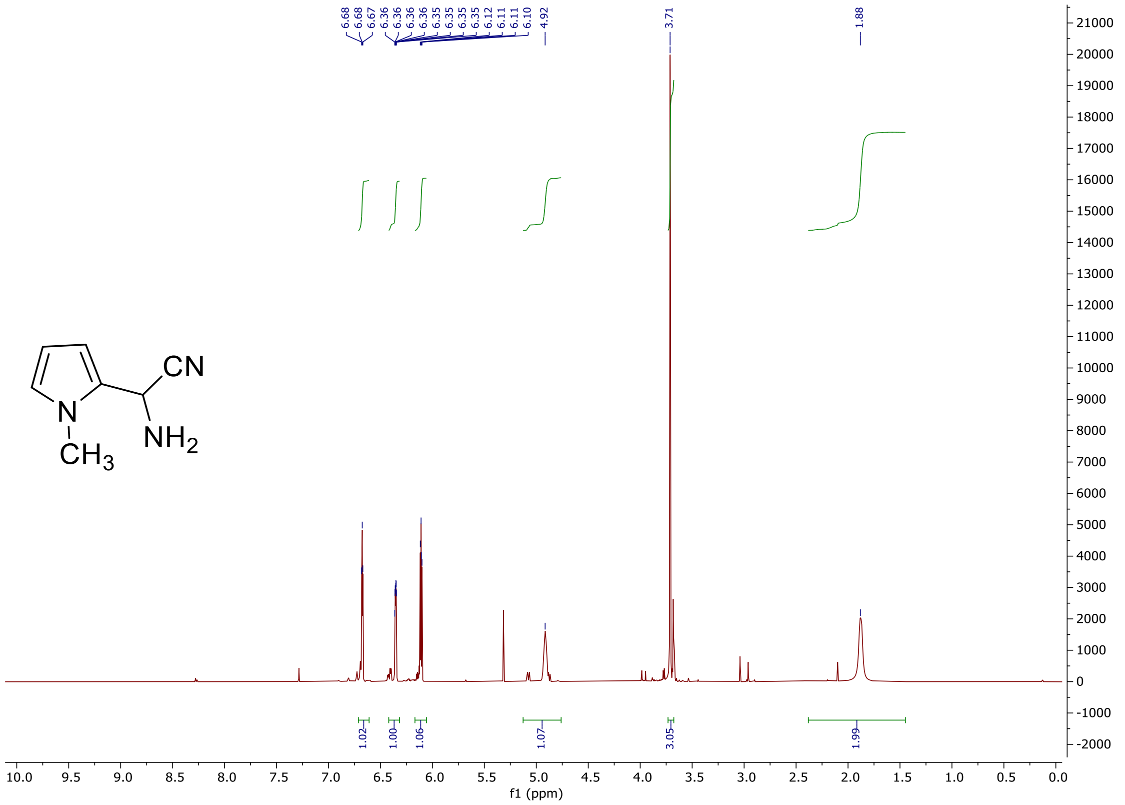

Figure S1. ^{1}H NMR spectrum ($CDCl_3$, 400 MHz, 296K) of α-Amino-1-methyl-1*H*-pyrrol-2-acetonitrile **3b**.

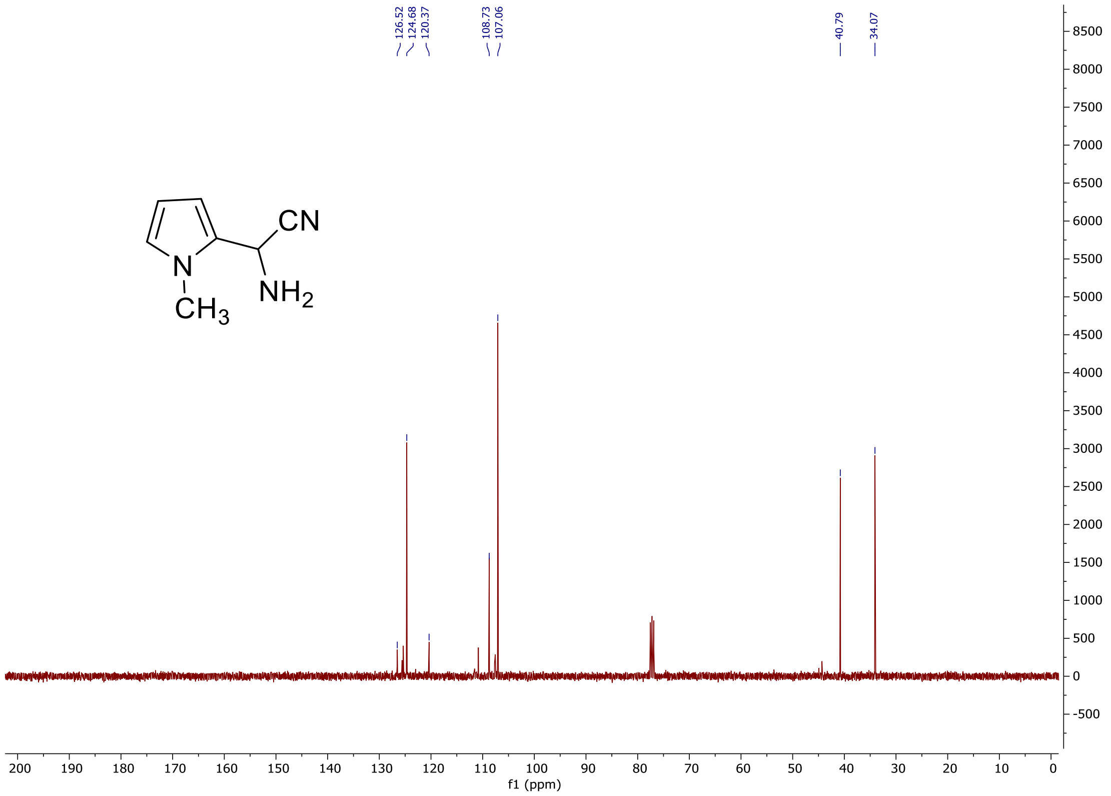

Figure S2. ^{13}C NMR spectrum ($CDCl_3$, 100 MHz, 296K) of α-Amino-1-methyl-1*H*-pyrrol-2-acetonitrile **3b**.

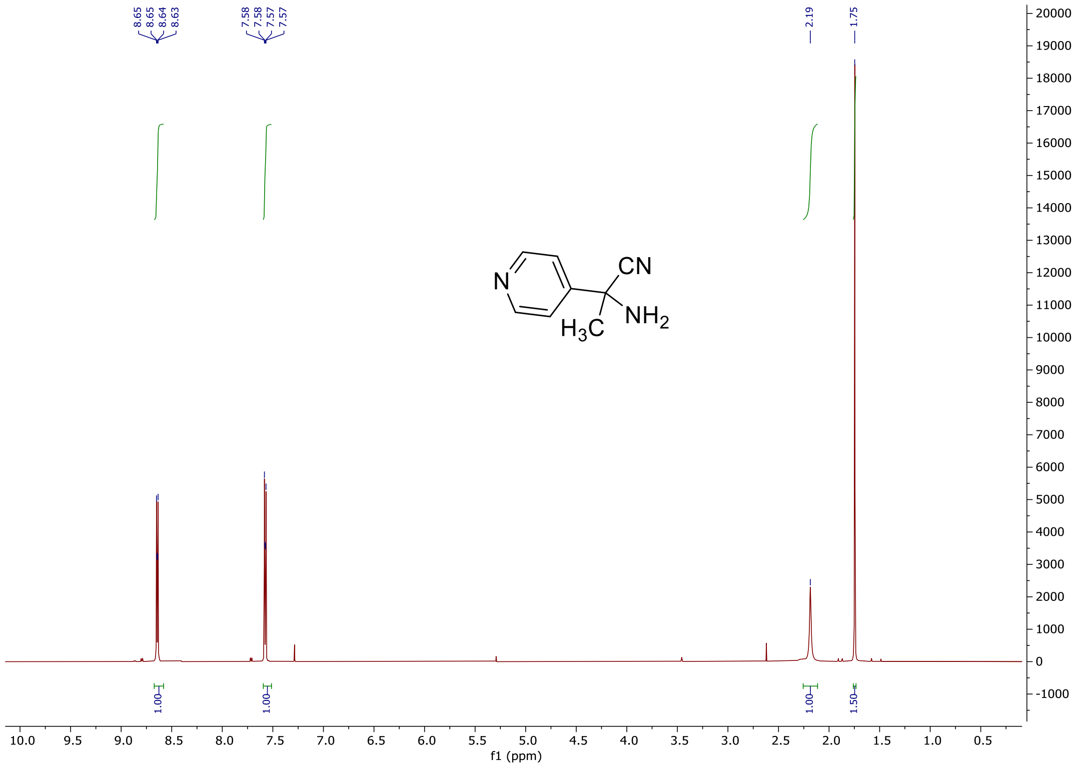

Figure S3. ^{1}H NMR spectrum ($CDCl_3$, 400 MHz, 296K) of α-Amino-α-methyl-4-pyridinacetonitrile **4f.**

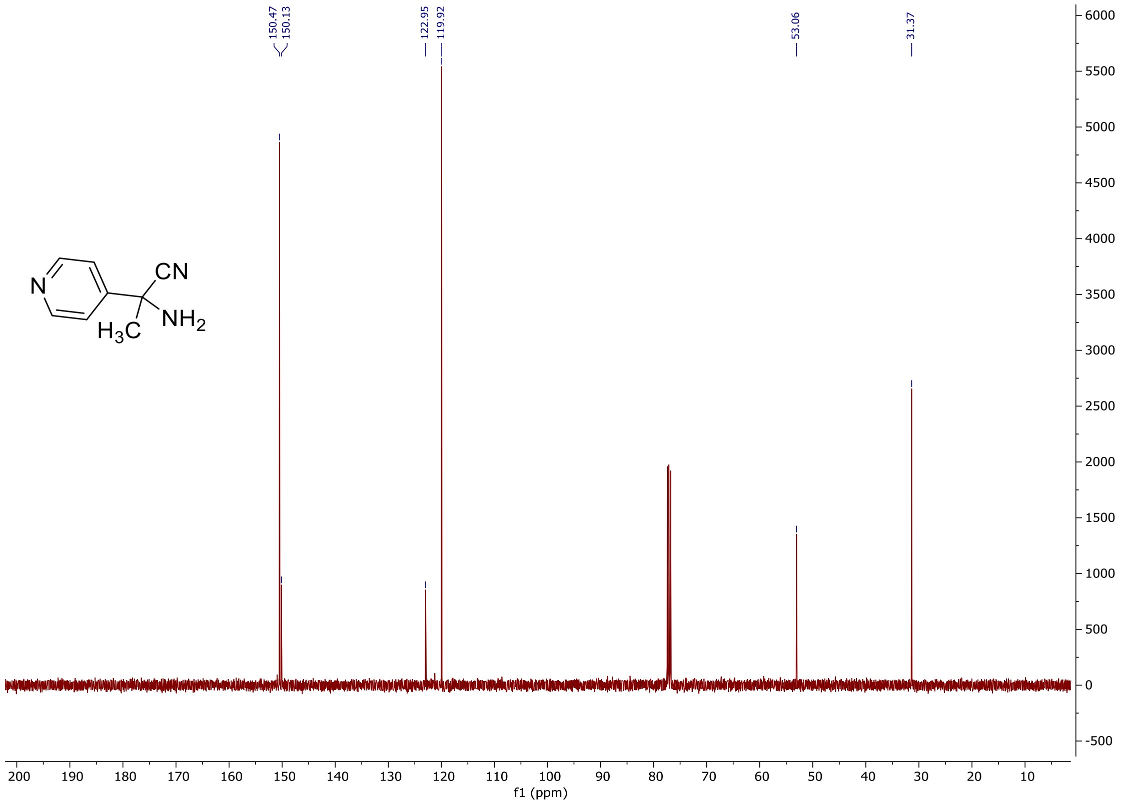

Figure S4. ^{13}C NMR spectrum ($CDCl_3$, 100 MHz, 296K) of α-Amino-α-methyl-4-pyridinacetonitrile **4f**.

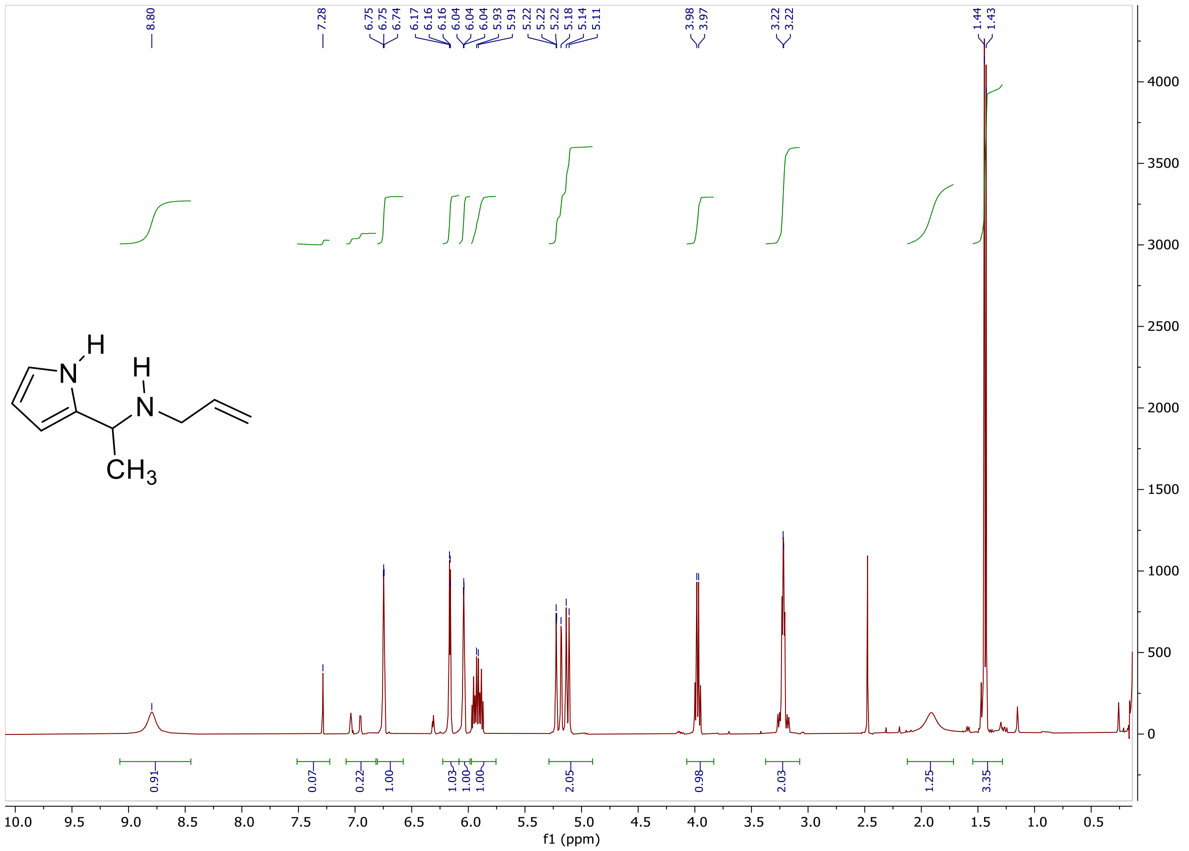

Figure S5. ^{1}H NMR spectrum ($CDCl_3$, 400 MHz, 296K) of α-Methyl-*N*-2-Propen-1-yl-1*H*-pyrrole-2-methanamine **5b.**

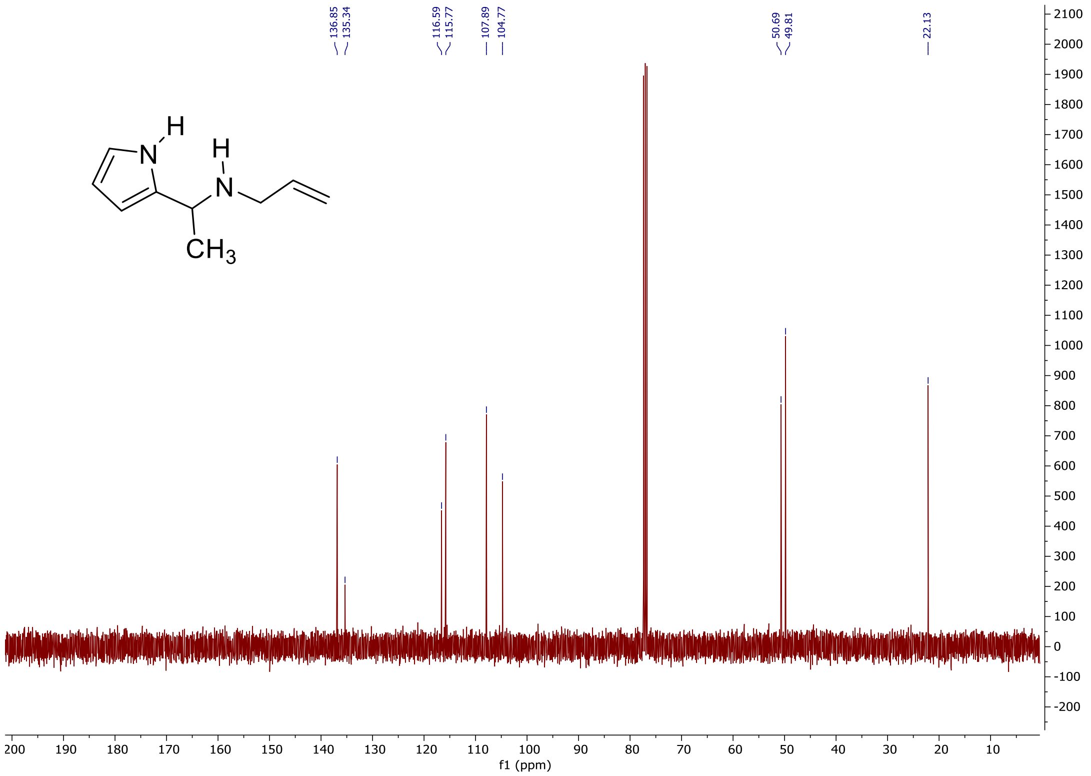

Figure S6. ^{13}C NMR spectrum ($CDCl_3$, 100 MHz, 296K) of α-Methyl-*N*-2-Propen-1-yl-1*H*-pyrrole-2-methanamine **5b.**

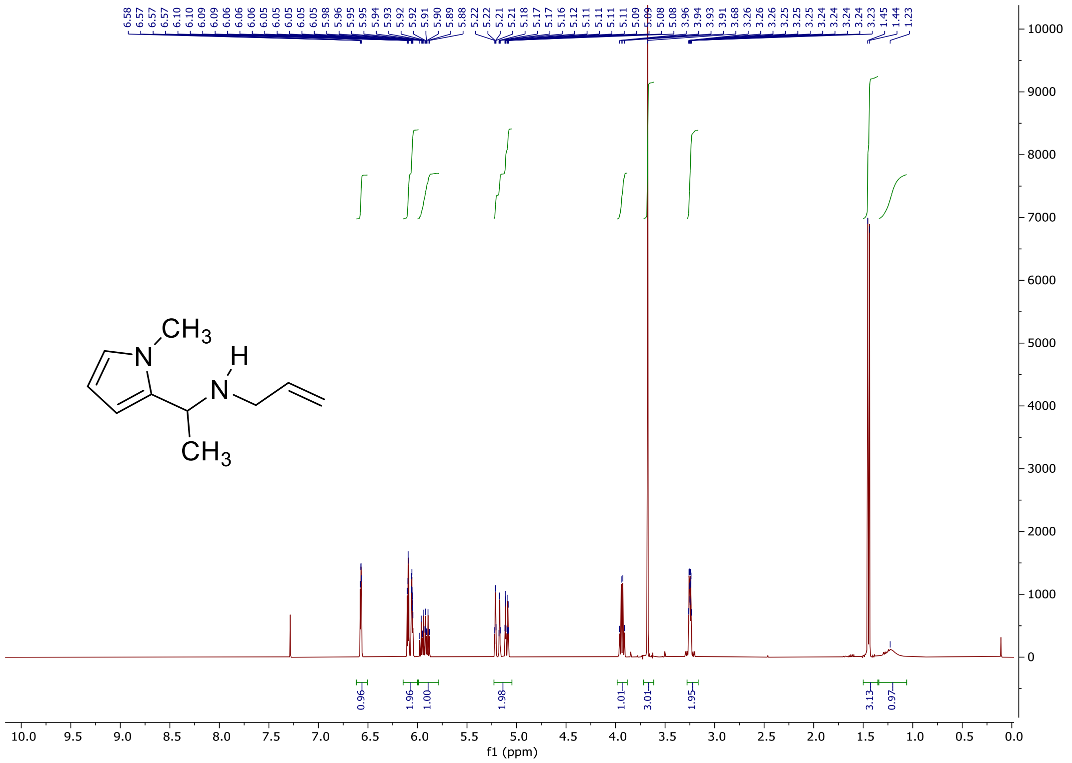

Figure S7. ^{1}H NMR spectrum ($CDCl_3$, 400 MHz, 296K) of α-Methyl-1-methyl-*N*-2-propen-1-yl-1*H*-pyrrole-2-methanamine **5d**.

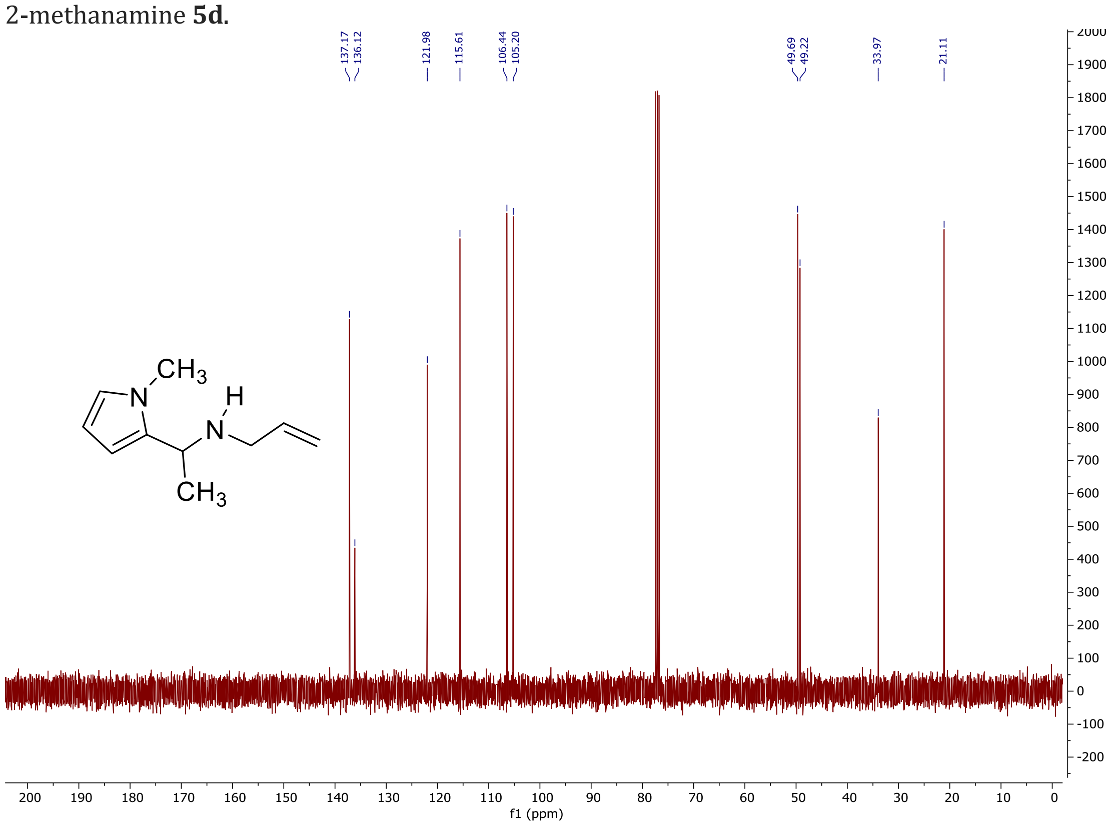

Figure S8. ^{13}C NMR spectrum ($CDCl_3$, 100 MHz, 296K) of α-Methyl-1-methyl-*N*-2-propen-1-yl-1*H*-pyrrole-2-methanamine **5d.**

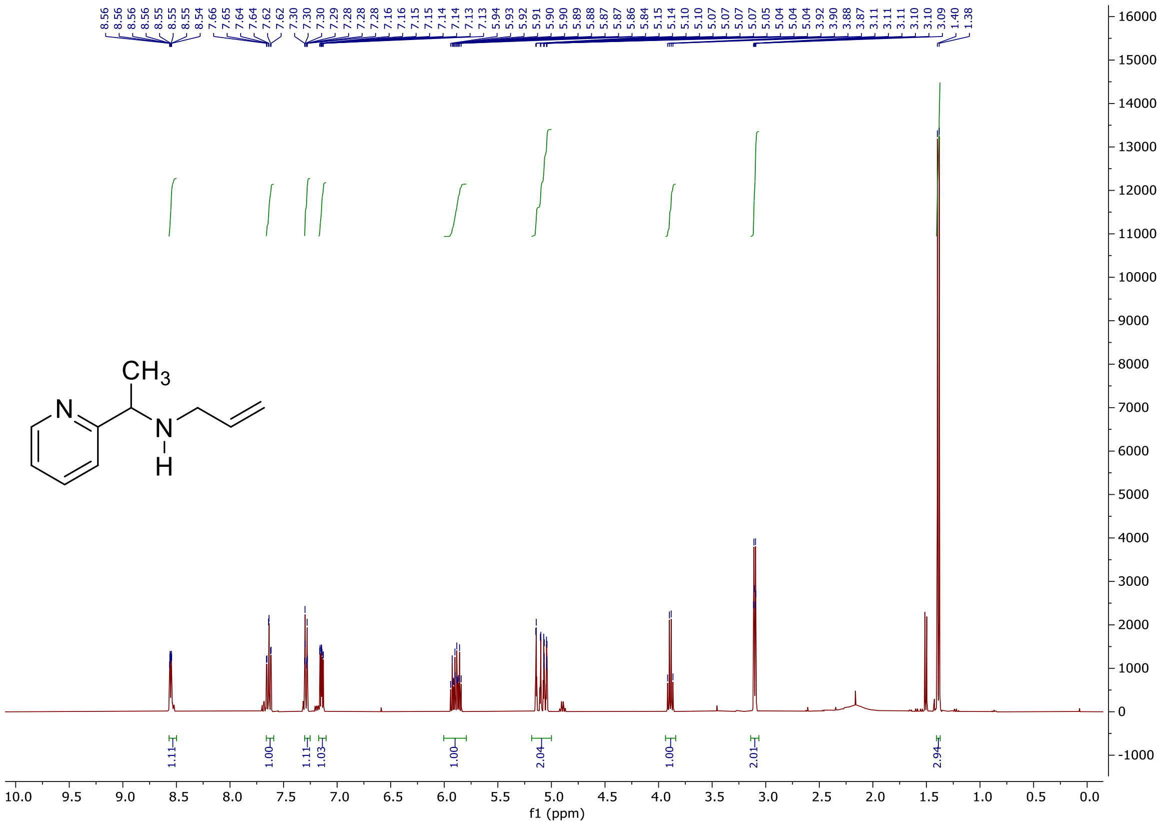

Figure S9. ^{1}H NMR spectrum ($CDCl_3$, 400 MHz, 296K) of α-Methyl-*N*-2-propen-1-yl-2-pyridinemethanamine **6b.**

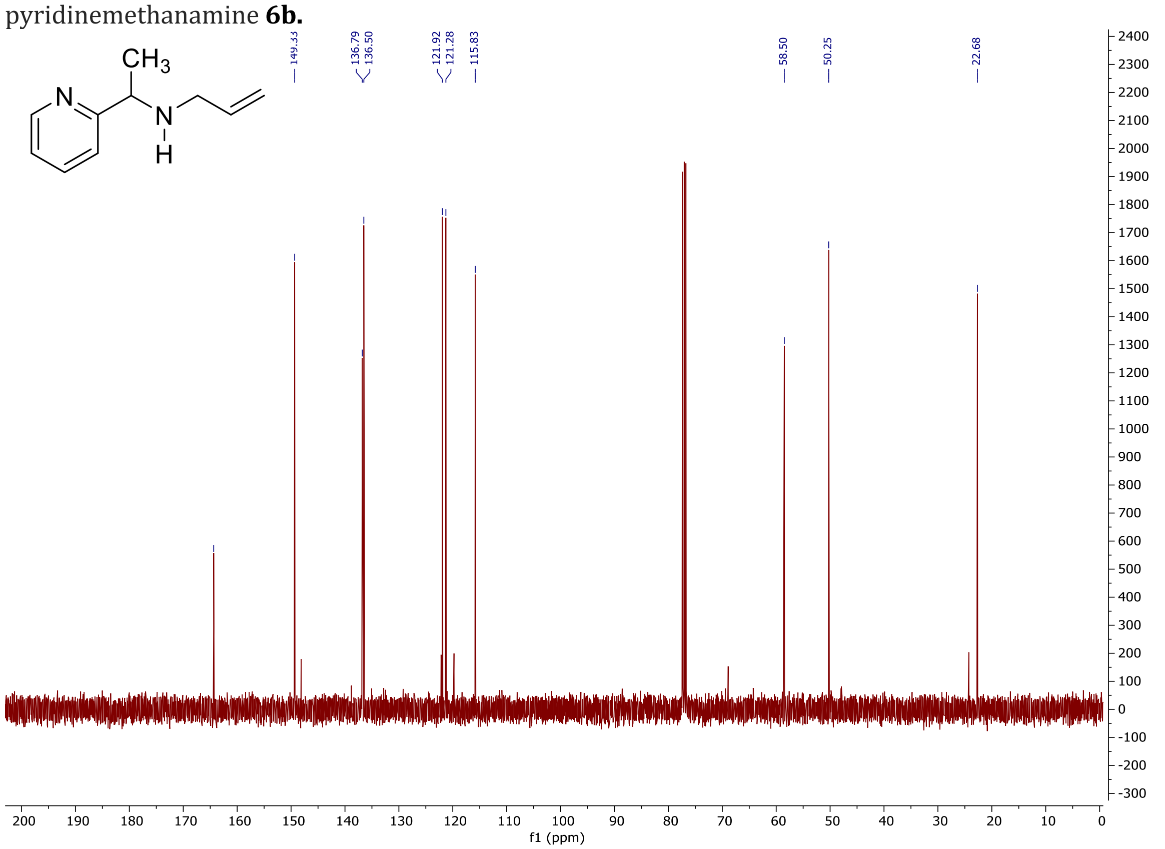

Figure S10. ^{13}C NMR spectrum ($CDCl_3$, 100 MHz, 296K) of α-Methyl-*N*-2-propen-1-yl-2-pyridinemethanamine **6b.**

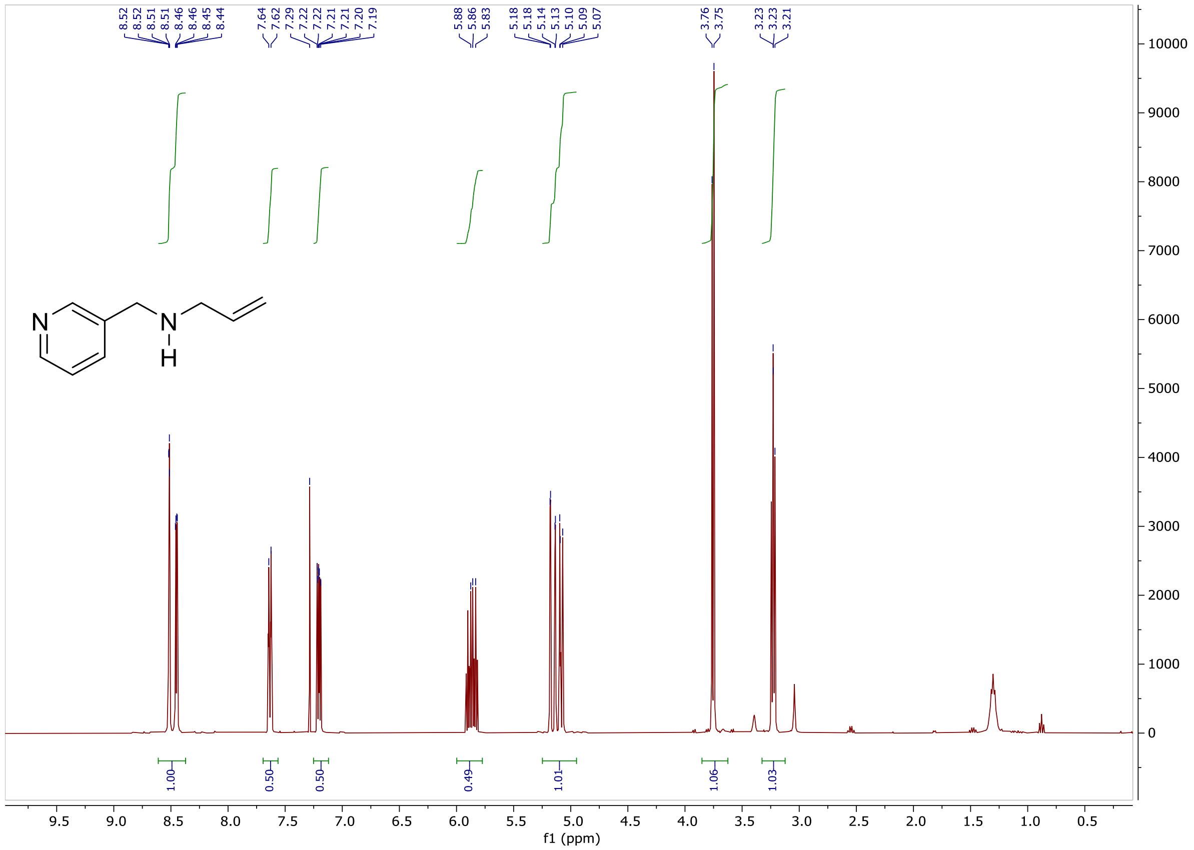

Figure S11. ^{1}H NMR spectrum ($CDCl_3$, 400 MHz, 296K) of *N*-2-Propen-1-yl-3-pyridinemethanamine **6c.**

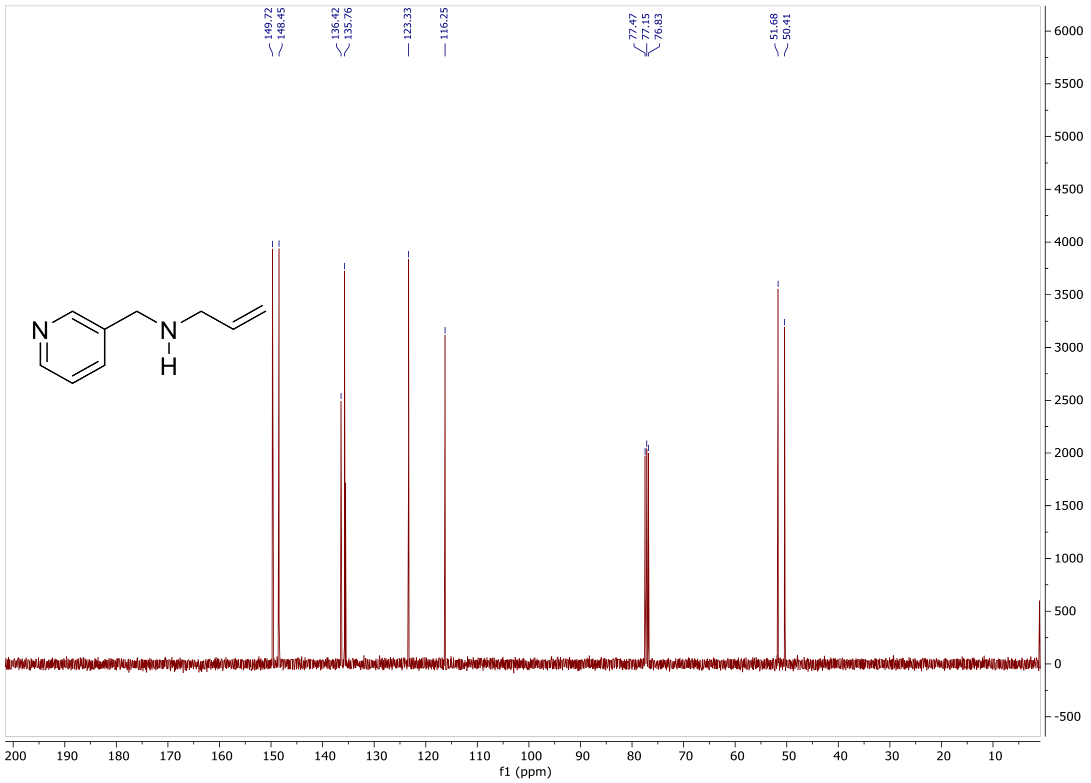

Figure S12. ^{13}C NMR spectrum ($CDCl_3$, 100 MHz, 296K) of *N*-2-Propen-1-yl-3-pyridinemethanamine **6c**

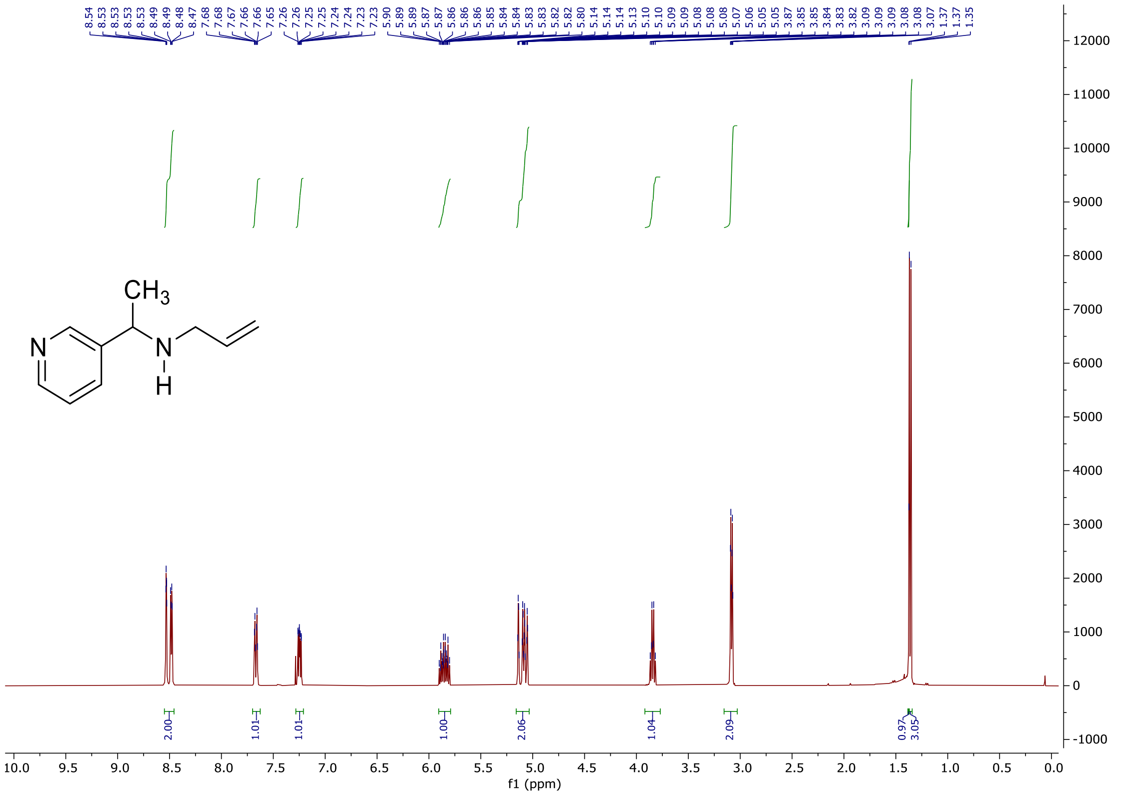

Figure S13. ^{1}H NMR spectrum ($CDCl_3$, 400 MHz, 296K) of α-Methyl-*N*-2-propen-1-yl-3-pyridinemethanamine **6d.**

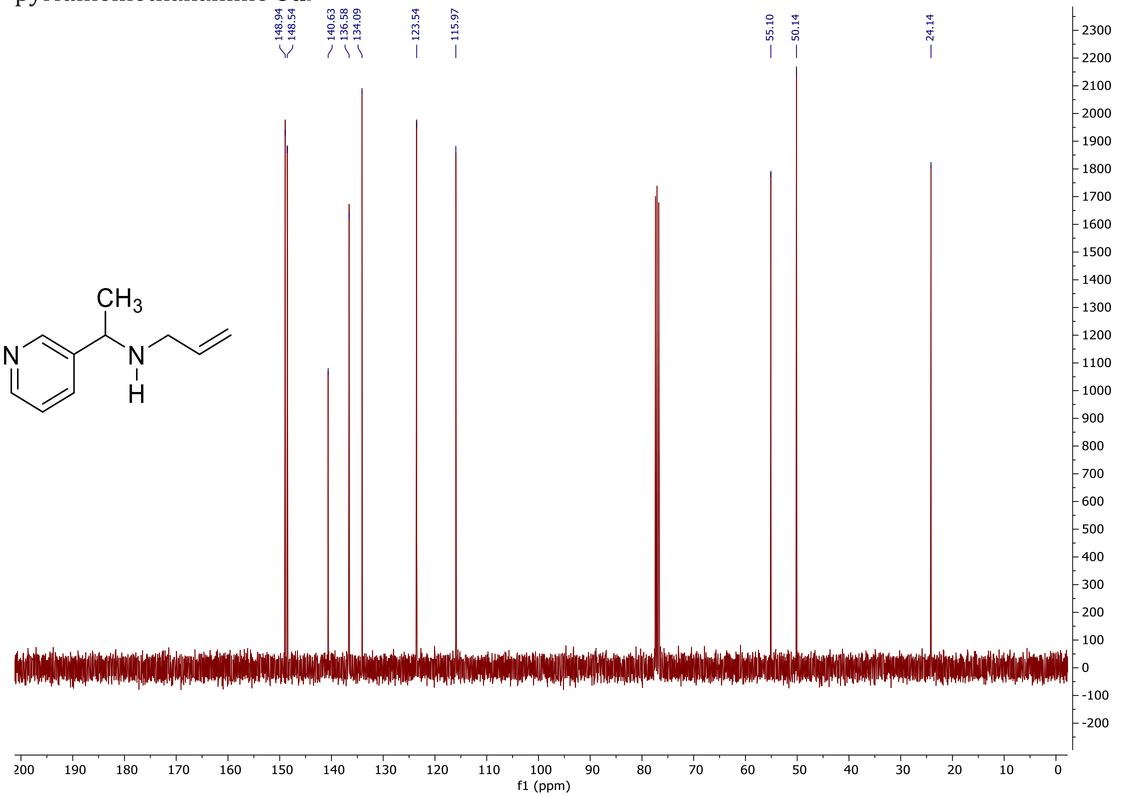

Figure S14. ^{13}C NMR spectrum ($CDCl_3$, 100 MHz, 296K) of α-Methyl-*N*-2-propen-1-yl-3-pyridinemethanamine **6d.**

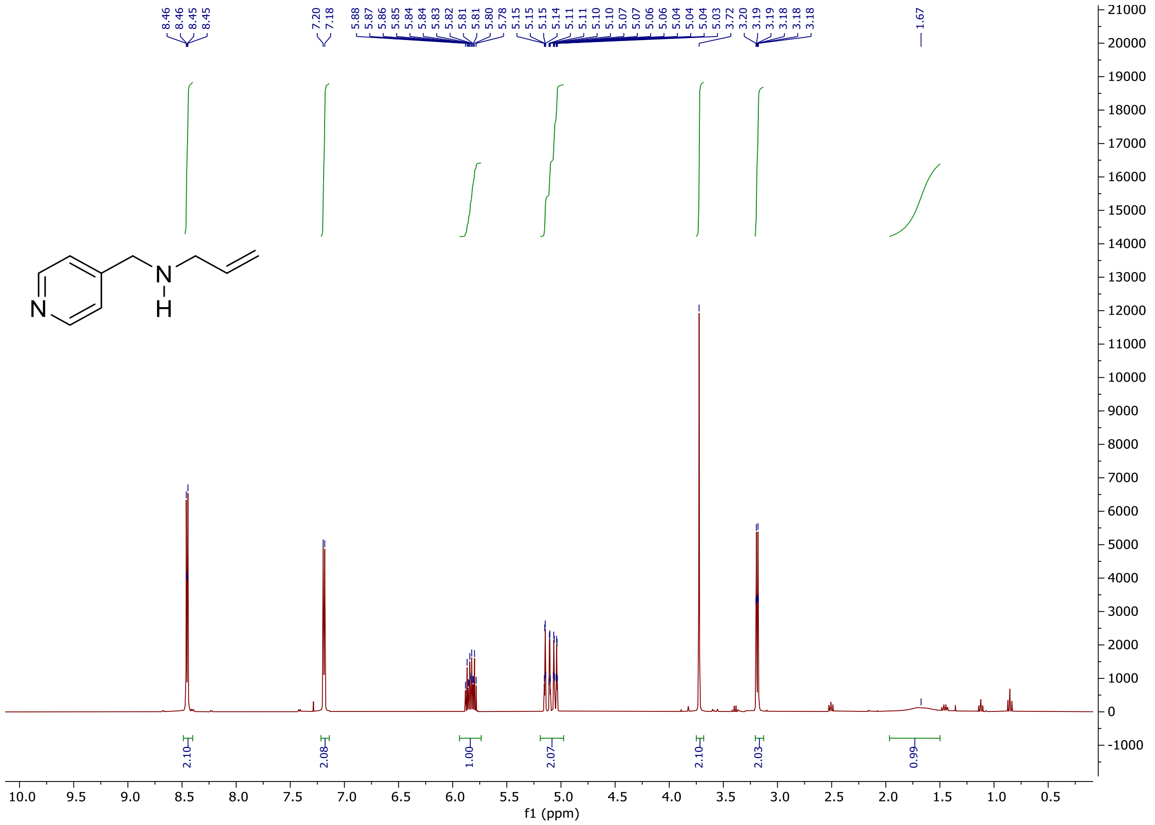

Figure S15. ¹H NMR spectrum ($CDCl_3$, 400 MHz, 296K) of *N*-2-Propen-1-yl-4-pyridinemethanamine **6e.**

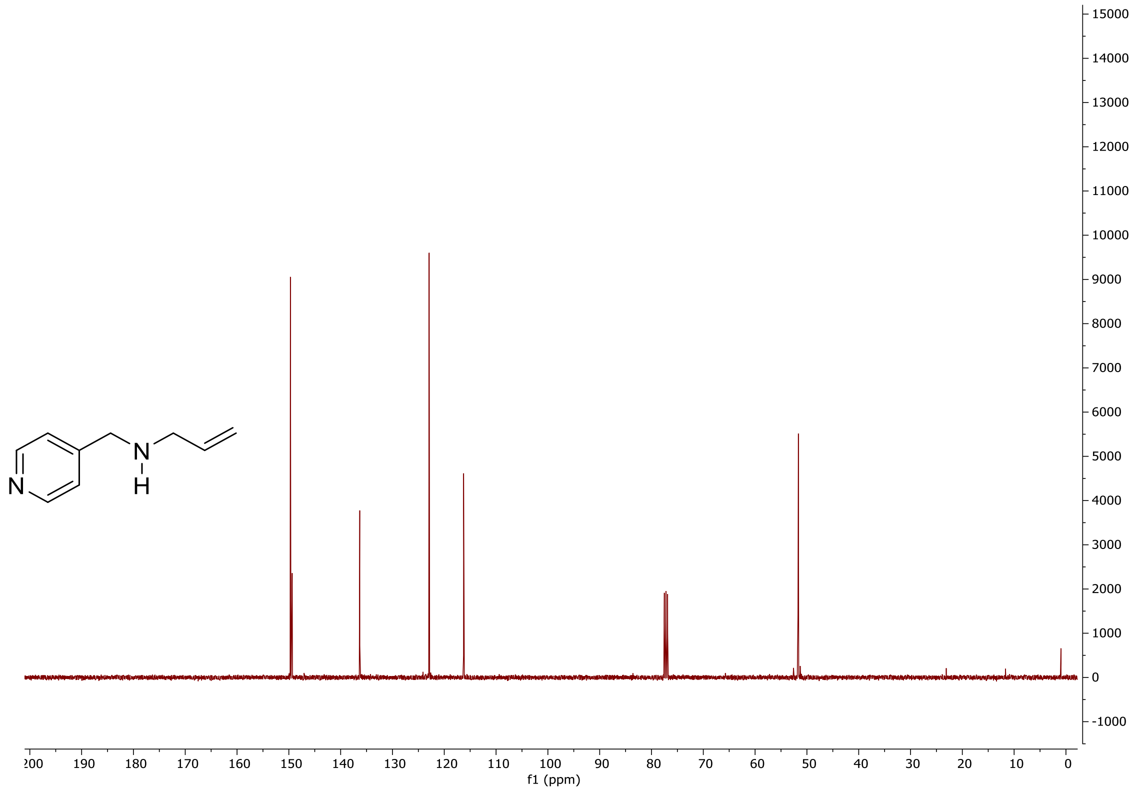

Figure S16. ¹³C NMR spectrum ($CDCl_3$, 100 MHz, 296K) of *N*-2-Propen-1-yl-4-pyridinemethanamine **6e.**

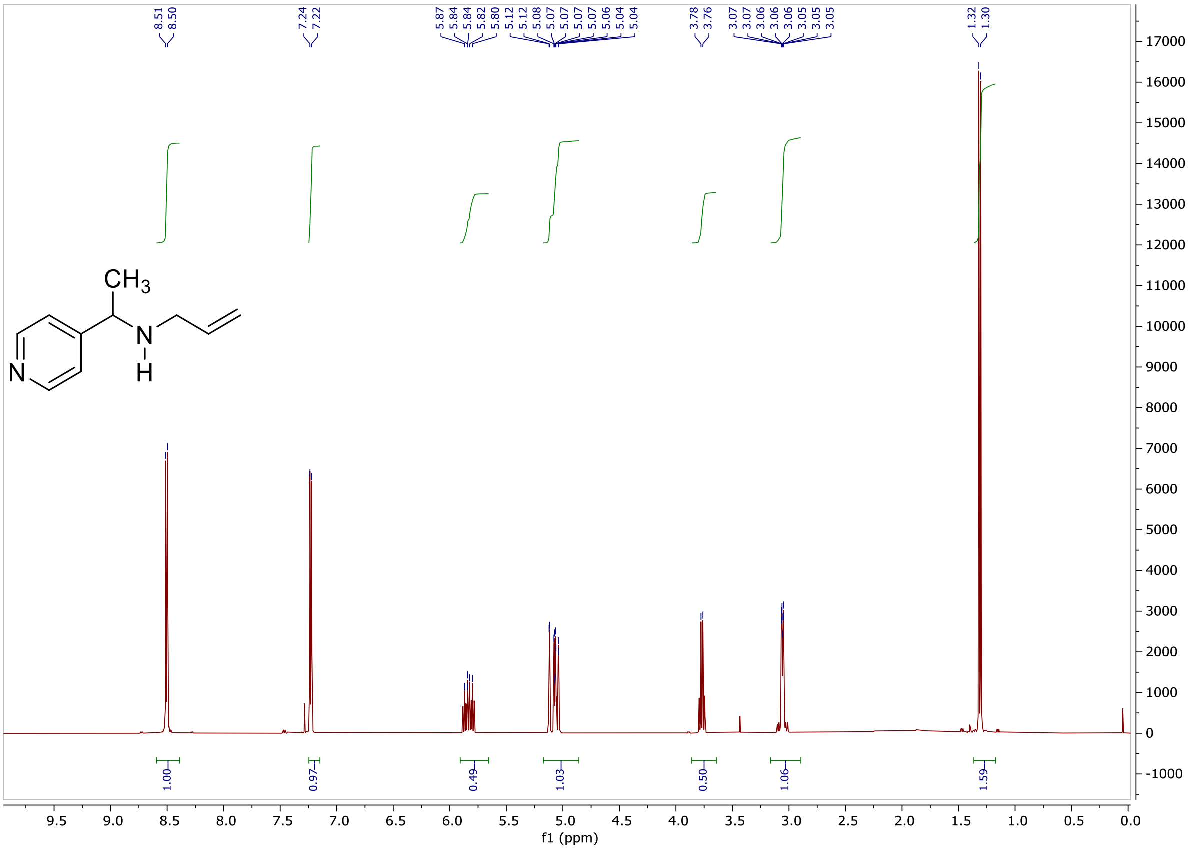

Figure S17. ^{1}H NMR spectrum ($CDCl_3$, 400 MHz, 296K) of α-Methyl-*N*-2-propen-1-yl-4-pyridinemethanamine **6f.**

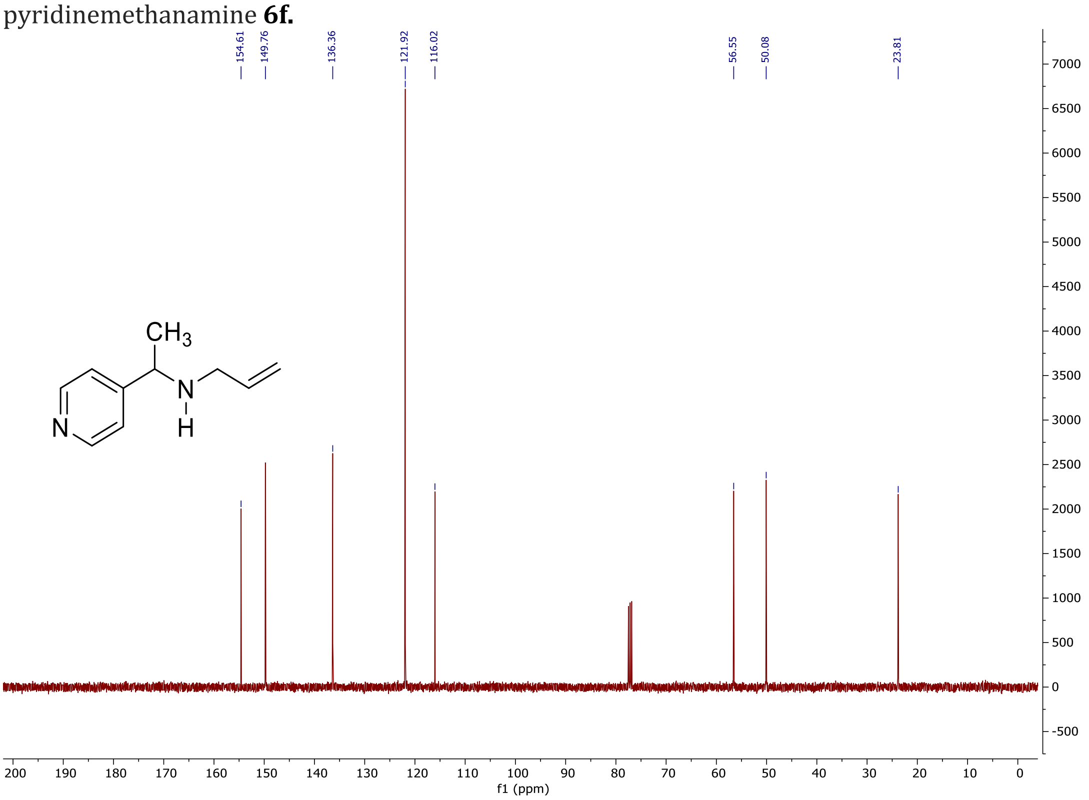

Figure S18. ^{13}C NMR spectrum ($CDCl_3$, 100 MHz, 296K) of α-Methyl-*N*-2-propen-1-yl-4-pyridinemethanamine **6f.**

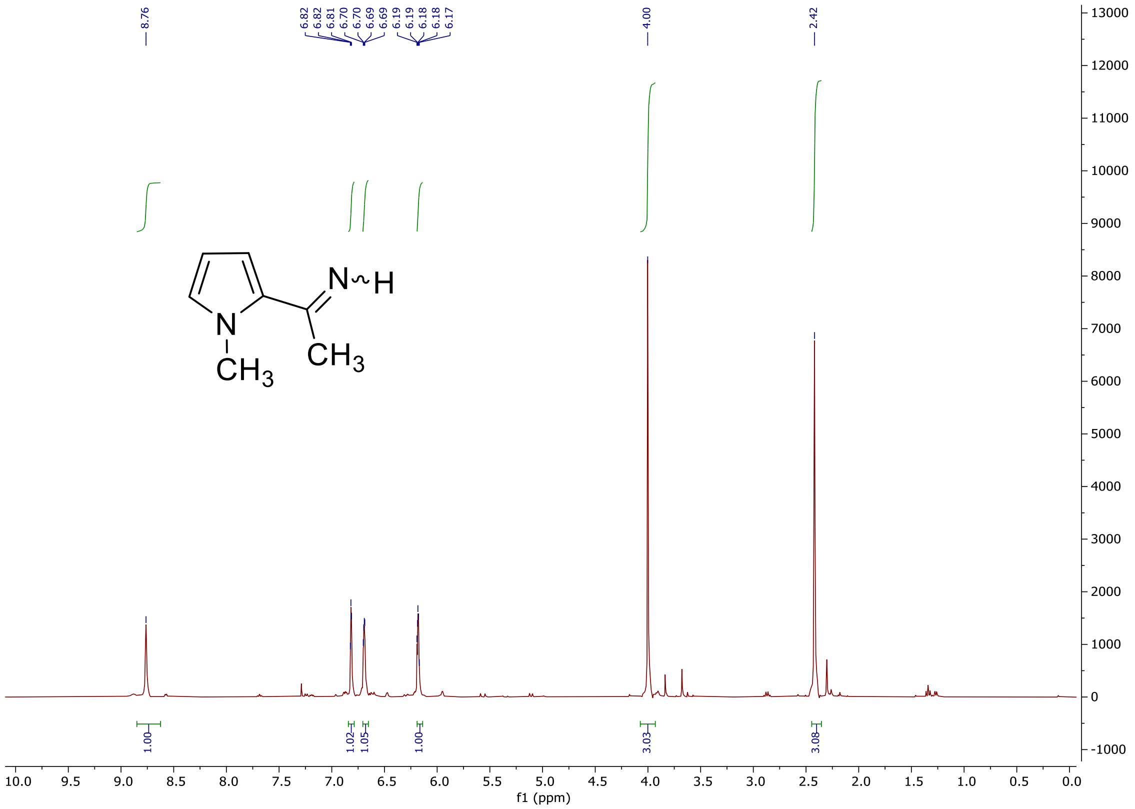

Figure S19. ¹H NMR spectrum (CDCl₃, 400 MHz, 223K) of N-Methyl-α-methyl-2-pyrrolemethanimine **1d.**

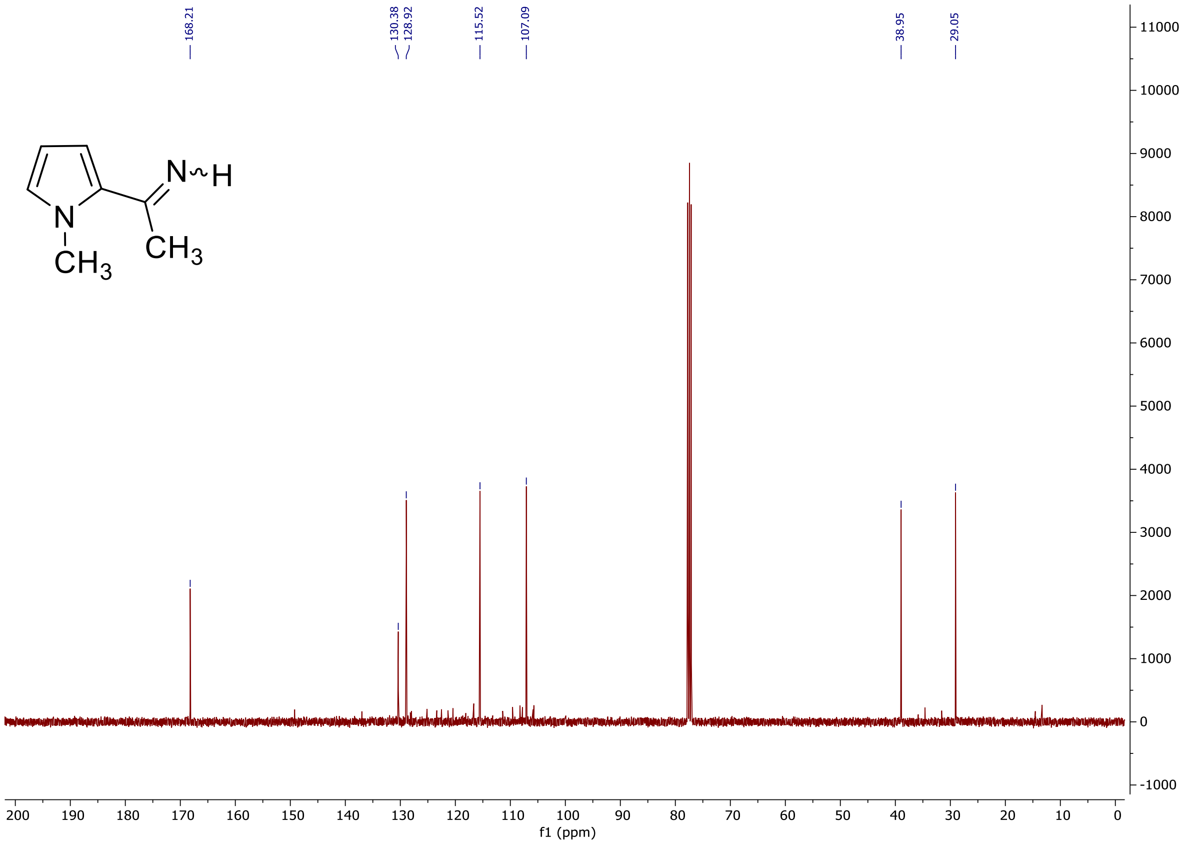

Figure S20. ¹³C NMR spectrum (CDCl₃, 100 MHz, 223K) of N-Methyl-α-methyl-2-pyrrolemethanimine **1d.**

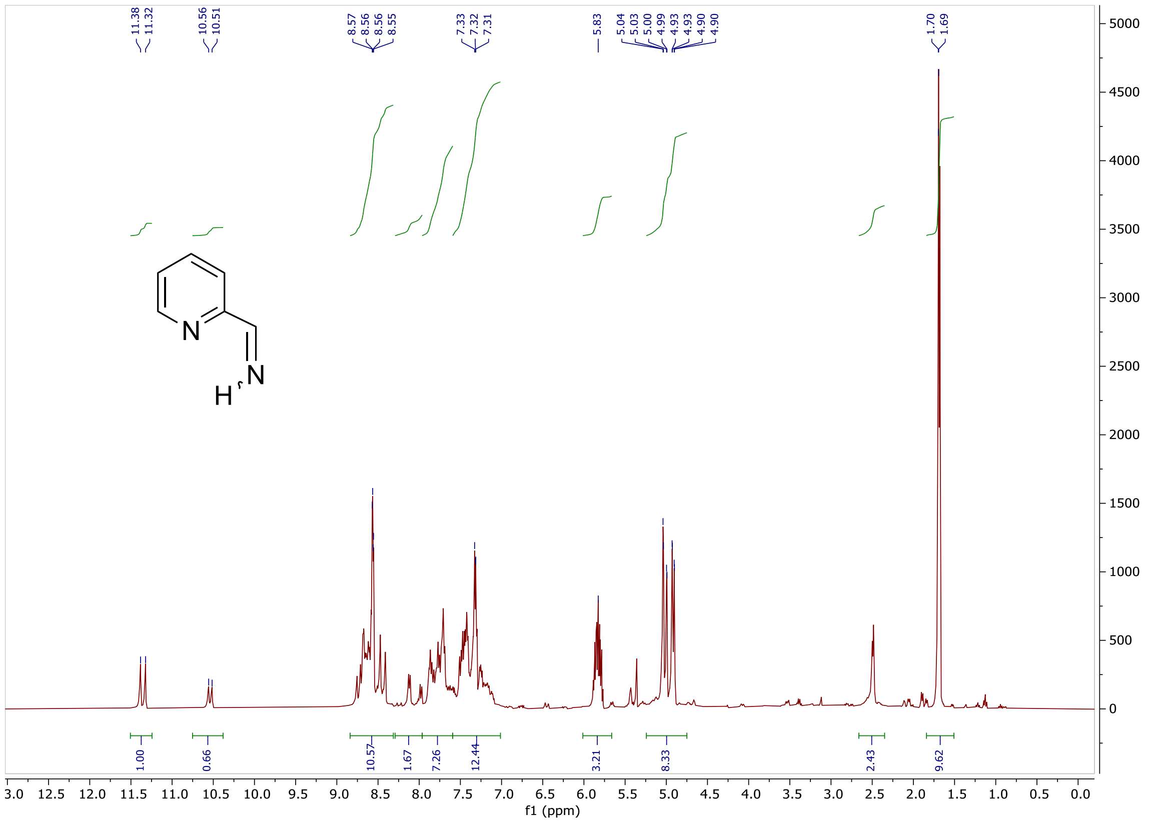

Figure S21. ^{1}H NMR spectrum (CD_2Cl_2, 400 MHz, 203K) of pyridine and propene (the main products of this thermolysis) and 2-pyridinemethanimine **2a.**

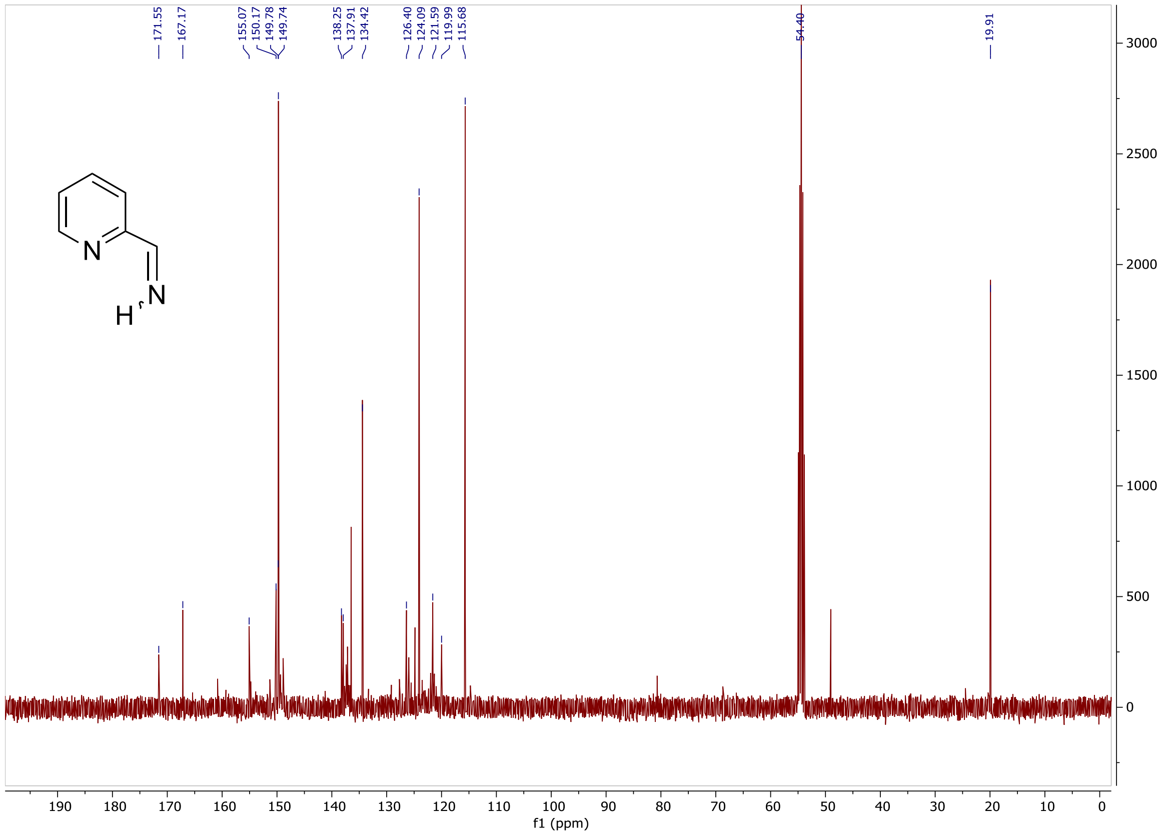

Figure S22. ^{13}C NMR spectrum (CD_2Cl_2, 100 MHz, 203K) of pyridine and propene (the main products of this thermolysis) and 2-pyridinemethanimine **2a.**

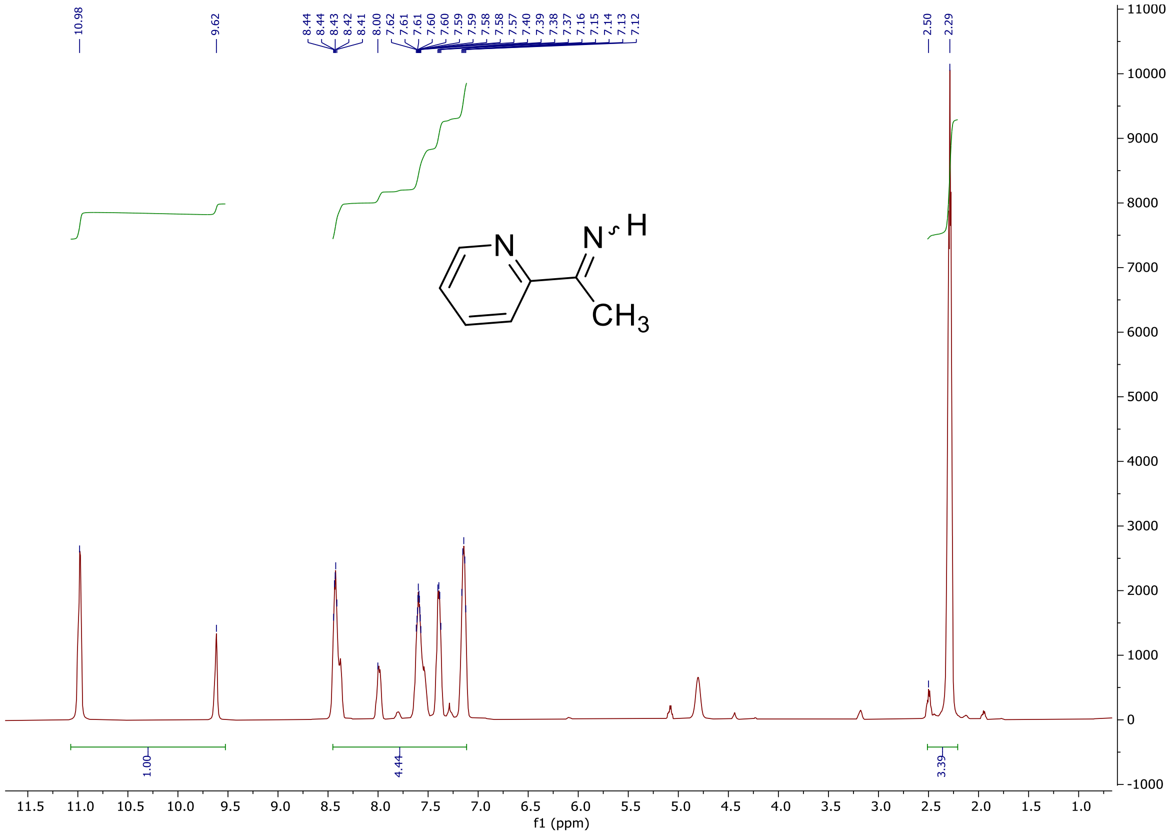

Figure S23. ^{1}H NMR spectrum ($CDCl_3$, 400 MHz, 223K) of α-Methyl-2-pyridinemethanimine **2b**.

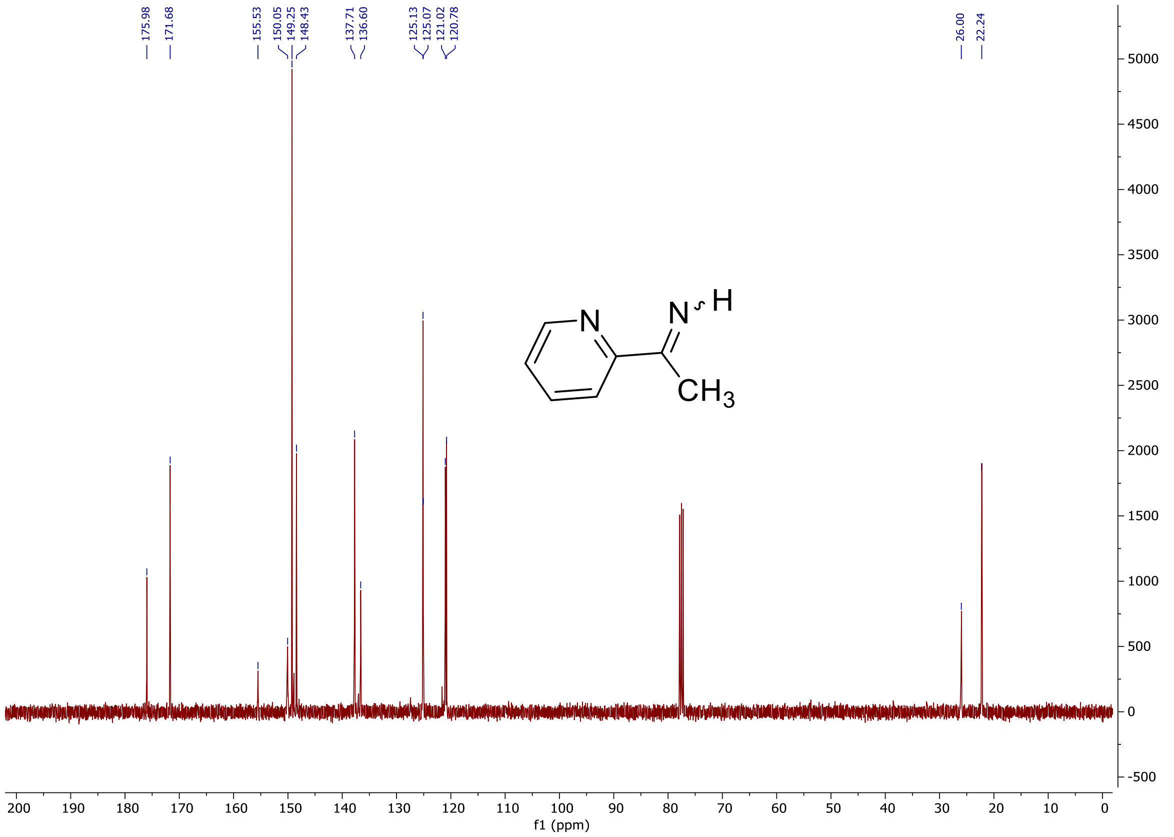

Figure S24. ^{13}C NMR spectrum ($CDCl_3$, 100 MHz, 223K) of α-Methyl-2-pyridinemethanimine **2b**.

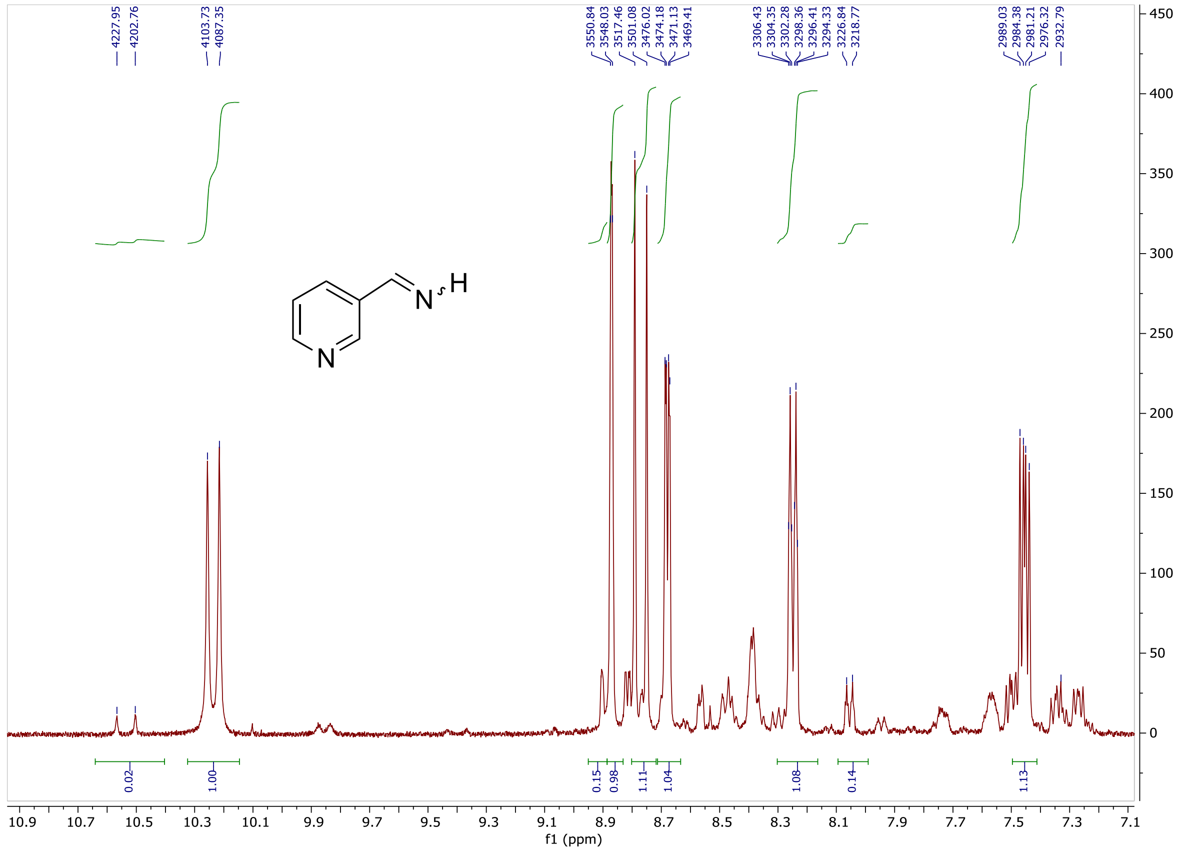

Figure S25. 7-11 ppm of ^{1}H NMR spectrum (CD_2Cl_2, 400 MHz, K) of 3-pyridinemethanimine **2c**.

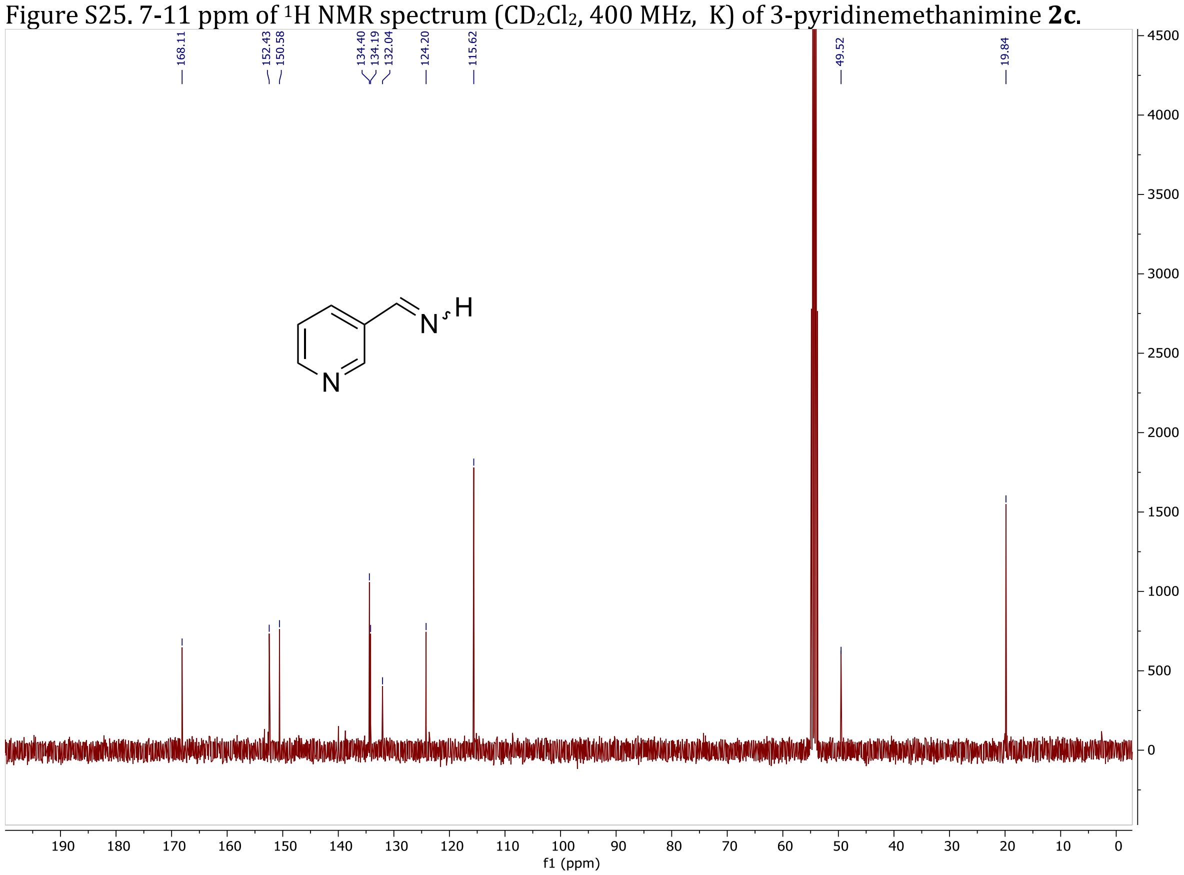

Figure S26. ^{13}C NMR spectrum (CD_2Cl_2, 100 MHz, 203K) of 3-pyridinemethanimine **2c** with propene.

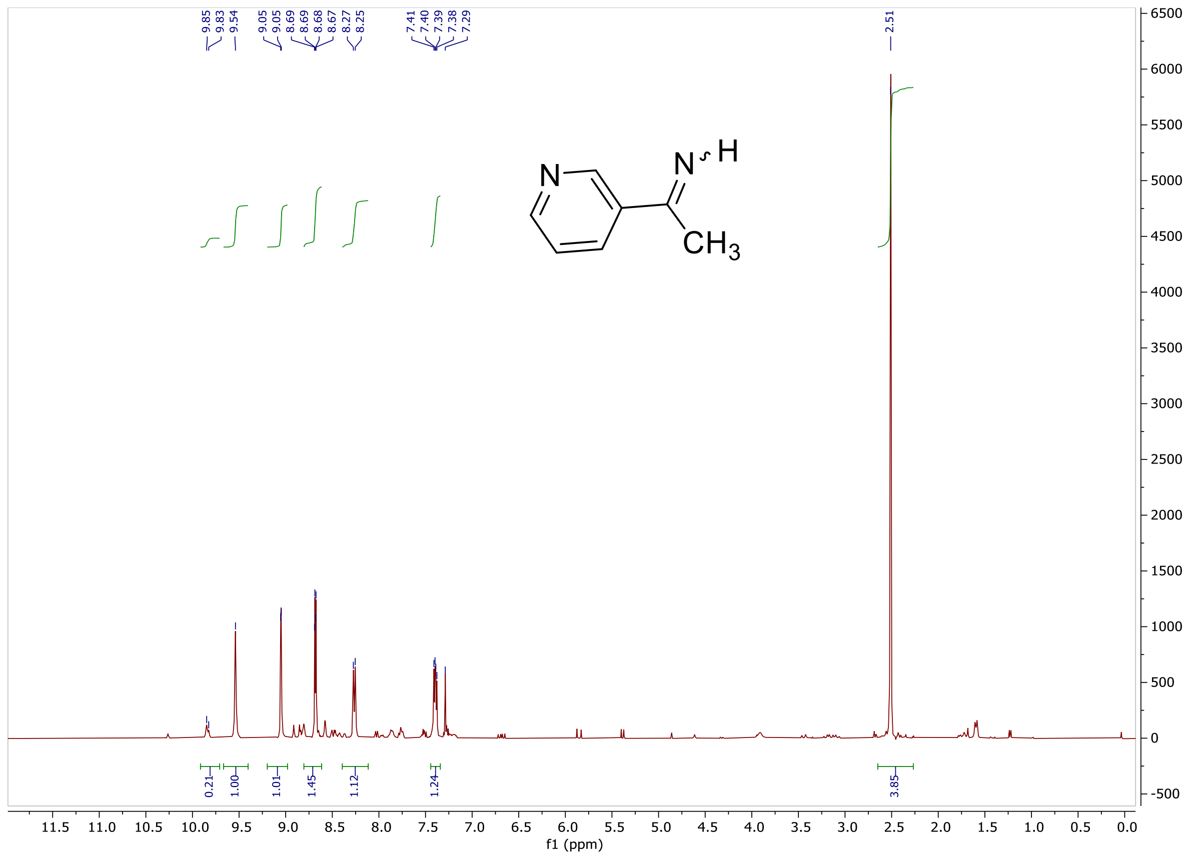

Figure S27. ^{1}H NMR spectrum ($CDCl_3$, 400 MHz, 233K) of α-Methyl-3-pyridinemethanimine **2d**.

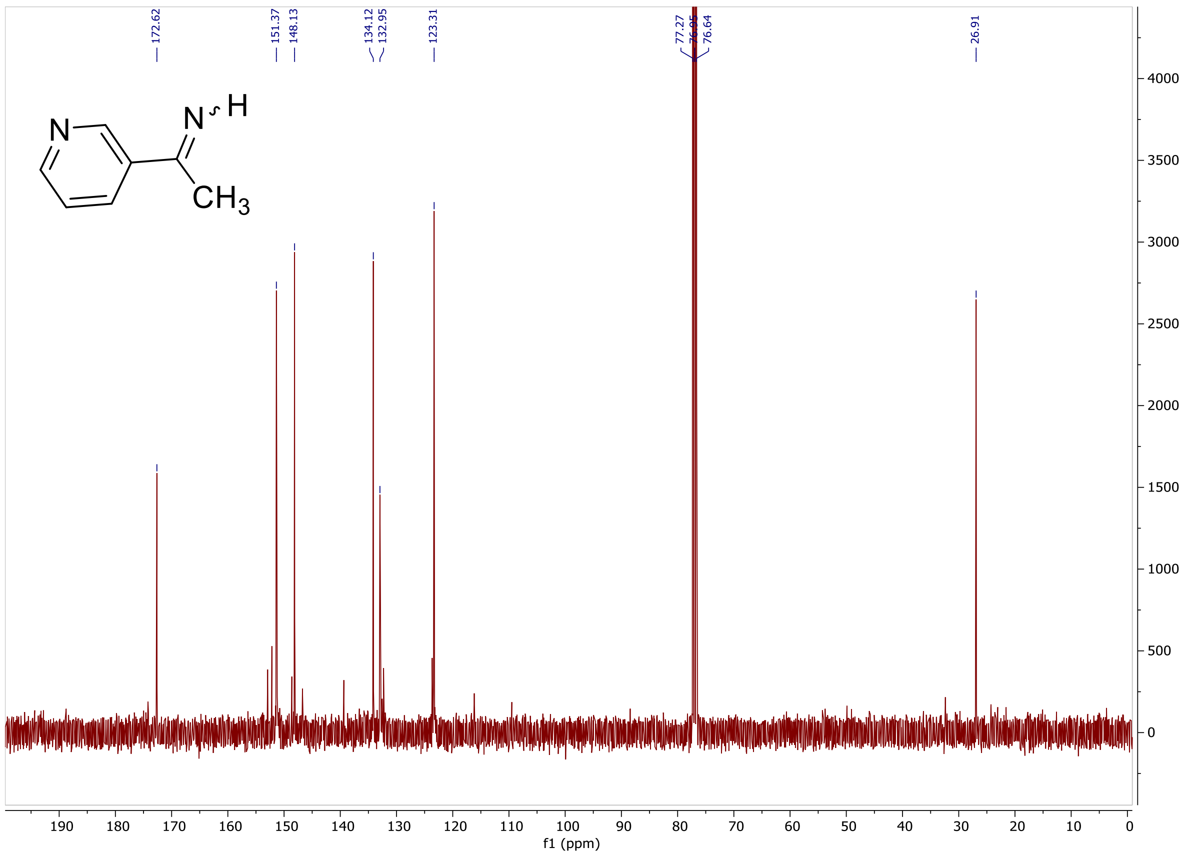

Figure S28. ^{13}C NMR spectrum ($CDCl_3$, 100 MHz, 223K) of α-Methyl-3-pyridinemethanimine **2d**.

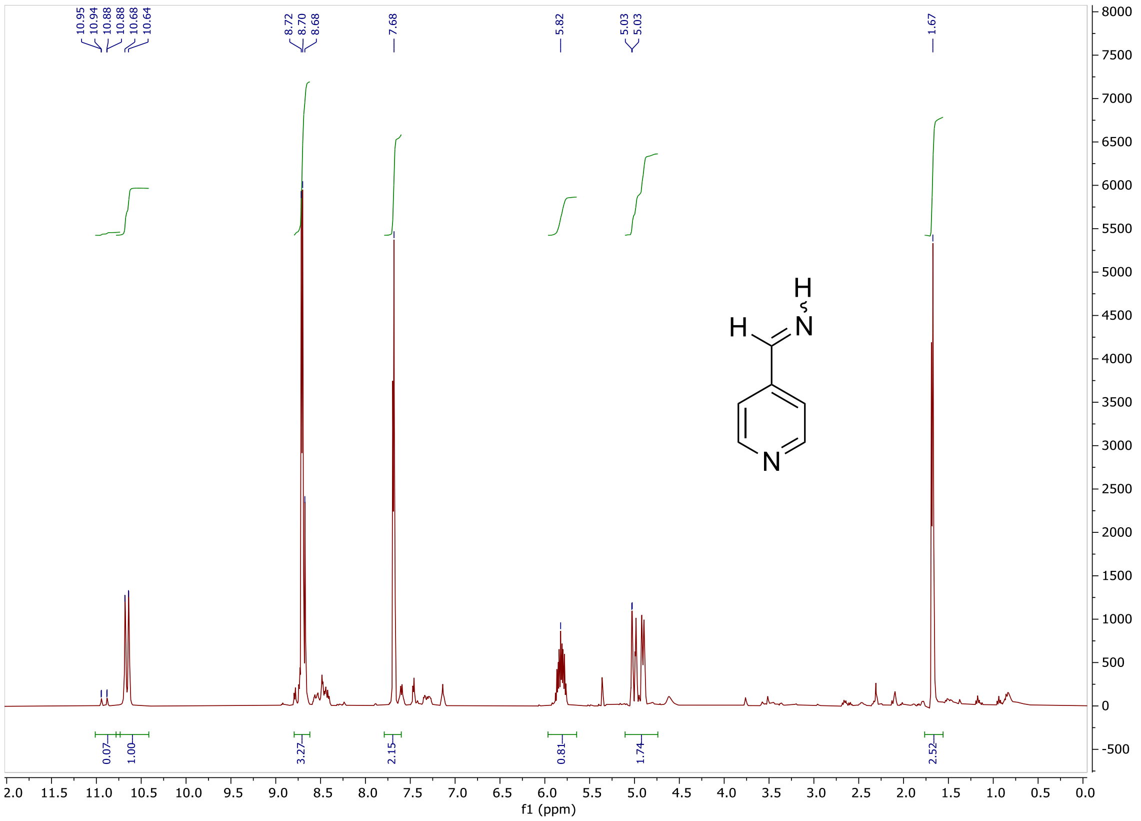

Figure S29. ^{1}H NMR spectrum (CD_2Cl_2, 400 MHz, 193K) of 4-pyridinemethanimine **2e**.

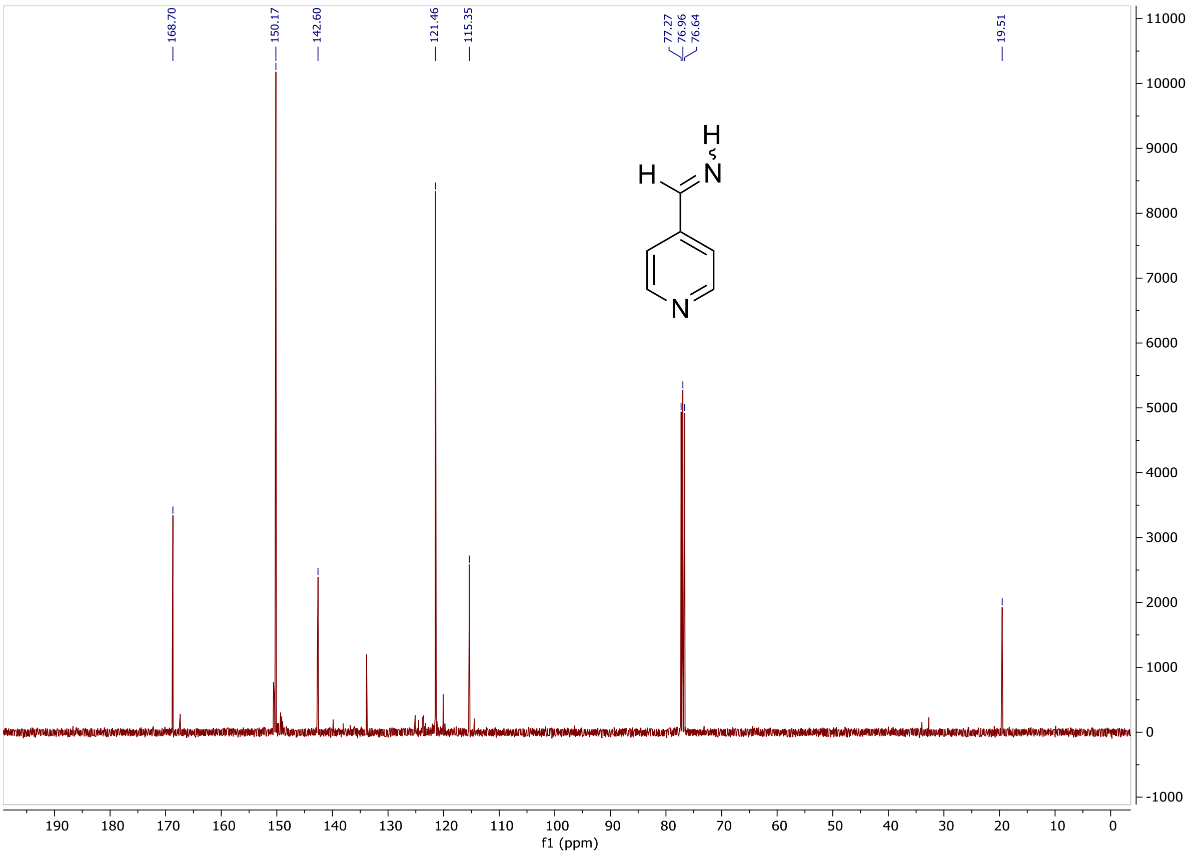

Figure S30. ^{13}C NMR spectrum ($CDCl_3$, 100 MHz, 223K) of 4-pyridinemethanimine **2e**.

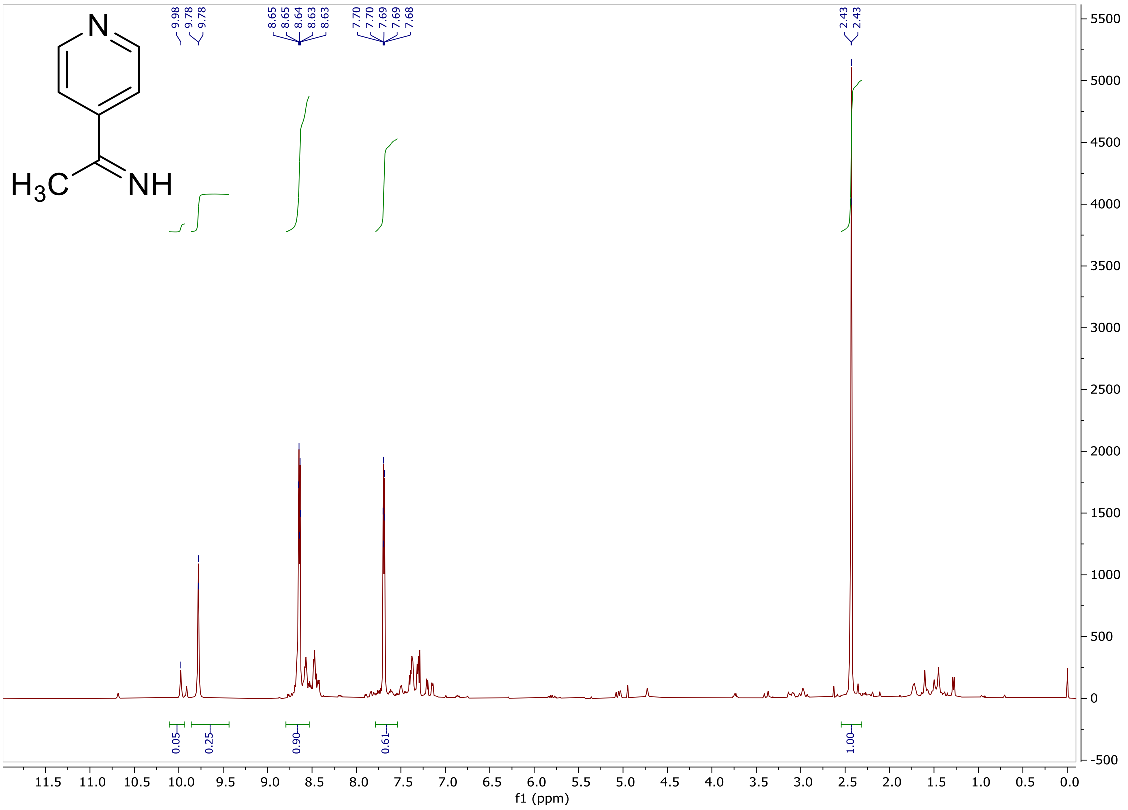

Figure S31. ^{1}H NMR spectrum ($CDCl_3$, 400 MHz, 223K) of α-Methyl-4-pyridinemethanimine **2f.**

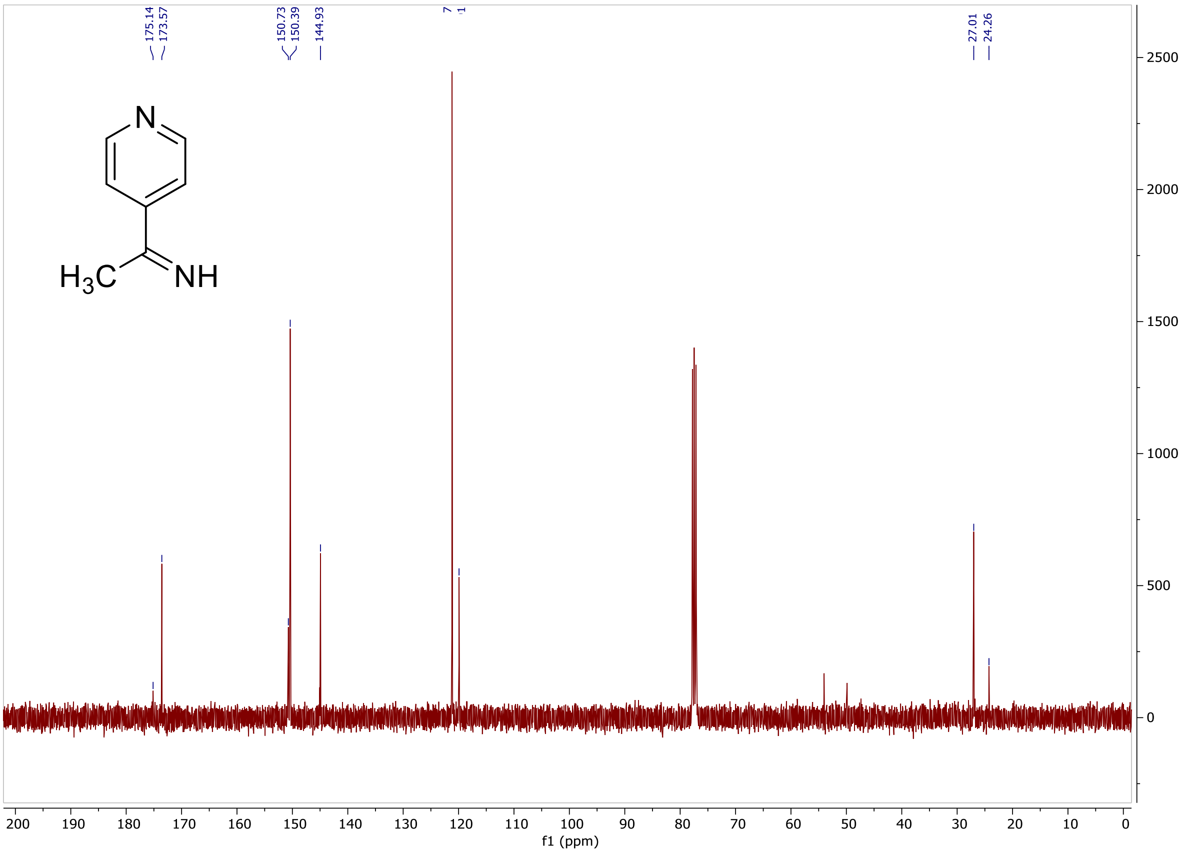

Figure S32. ^{13}C NMR spectrum ($CDCl_3$, 100 MHz, 223K) of α-Methyl-4-pyridinemethanimine **2f.**

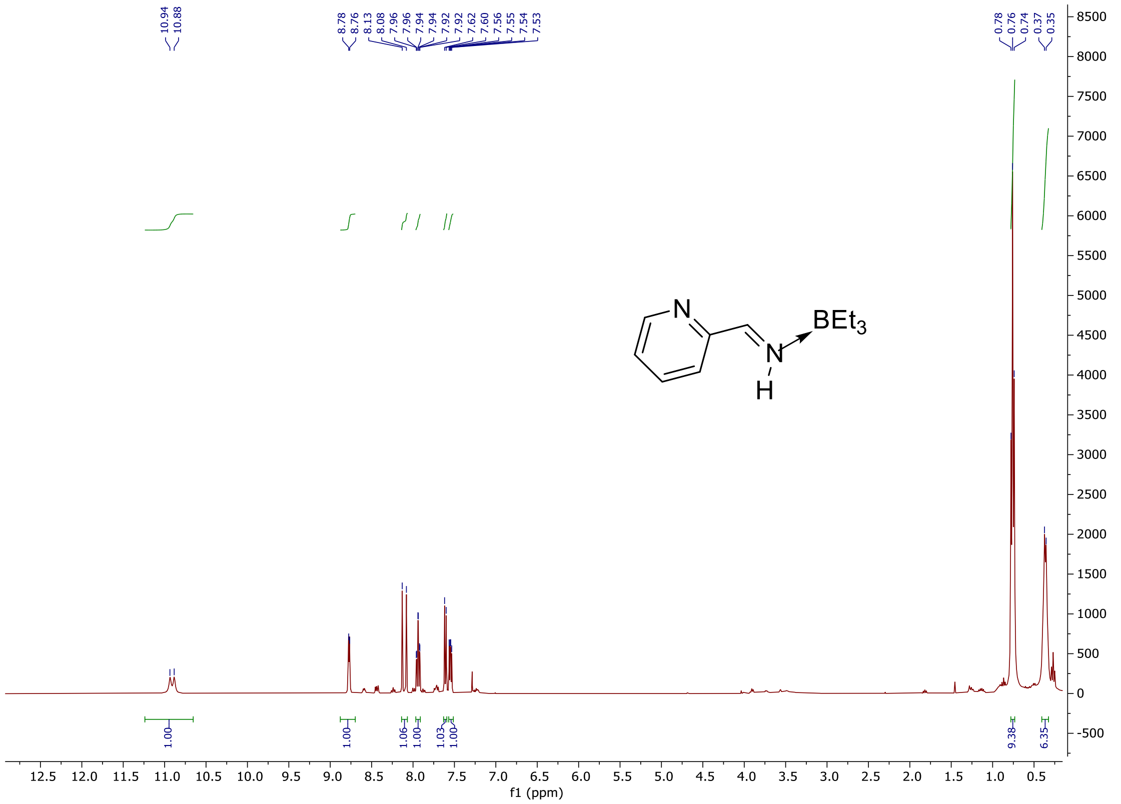

Figure S33. ^{1}H NMR spectrum ($CDCl_3$, 400 MHz, 296K) of (E)-2-Pyridinemethanimine-triethylborane complex **9a**.

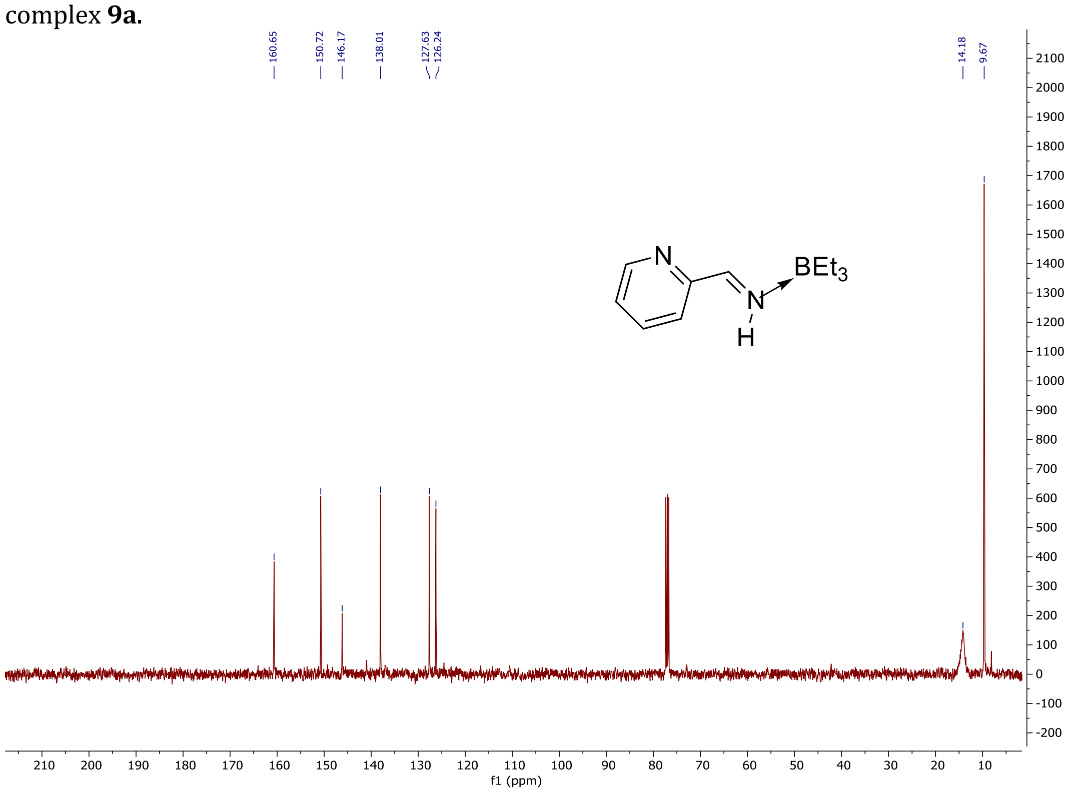

Figure S34. ^{13}C NMR spectrum ($CDCl_3$, 100 MHz, 296K) of (E)-2-Pyridinemethanimine-triethylborane complex **9a**.

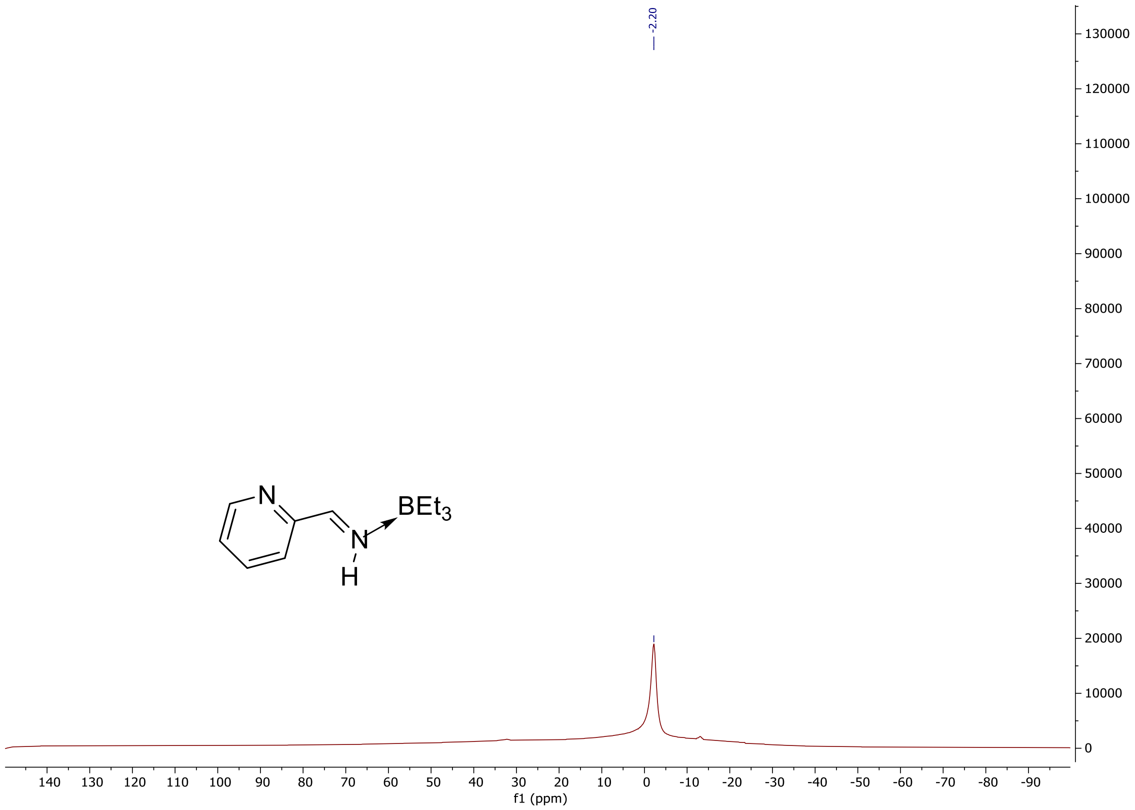

Figure 35. ^{11}B NMR spectrum ($CDCl_3$, 128 MHz, 296K) of (E)-2-Pyridinemethanimine-triethylborane complex **9a**.
